# Significance of Competitive Reactions in an Atmospheric Pressure Chemical Ionization Ion Source: Effect of Solvent

**DOI:** 10.1021/jasms.2c00034

**Published:** 2022-05-12

**Authors:** Younes Valadbeigi, Tim Causon

**Affiliations:** University of Natural Resources and Life Sciences, Vienna, Department of Chemistry, Institute of Analytical Chemistry, Muthgasse 18, 1190 Vienna, Austria

## Abstract

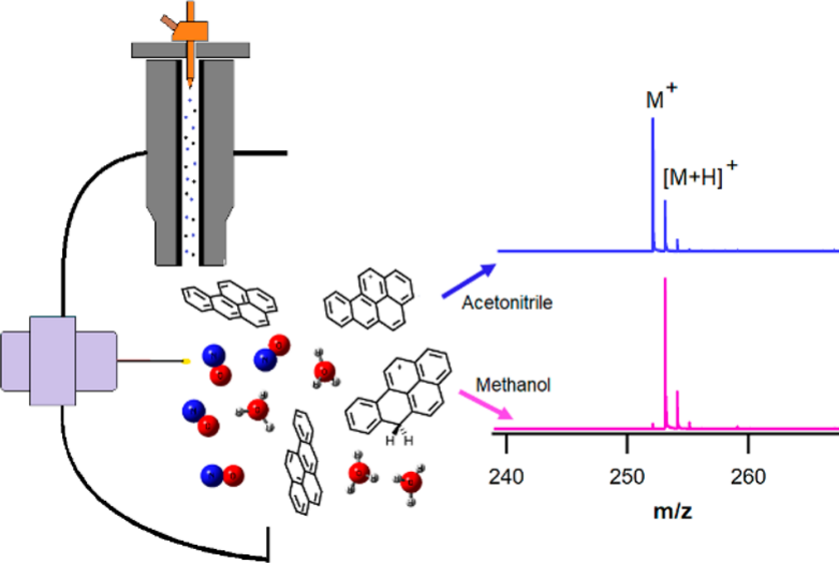

Ionization of organic compounds with different structural and energetic properties including benzene derivatives, polycyclic aromatic hydrocarbons (PAHs), ketones, and polyenes was studied using a commercial atmospheric pressure corona discharge (APCI) ion source on a drift tube ion mobility-quadrupole-time-of-flight mass spectrometer (IM-QTOFMS). It was found that the studied cohort of compounds can be experimentally ionized via protonation, charge transfer, and hydride abstraction leading to formation of [M + H]^+^, [M]^+•^, and [M – H]^+^ species, respectively. By experimentally monitoring the product ions and comparing the thermodynamic data for different ionization paths, it was proposed that NO^+^ is one of the main reactant ions (RIs) in the ion source used. Of particular focus in this work were theoretical and experimental studies of the effect of solvents frequently used for analytical applications with this ion source (acetonitrile, methanol, and chloroform) on the ionization mechanisms. In methanol, the studied compounds were observed to be ionized mainly via proton transfer while acetonitrile suppressed the protonation of compounds and enhanced their ionization via charge transfer and hydride abstraction. Use of chloroform as a solvent led to formation of CHCl_2_^+^ as an alternative reactant ion (RI) to ionize the analytes via electrophilic substitution. Density functional theory (DFT) was used to study the different paths of ionization. The theoretical and experimental results showed that by using only the absolute thermodynamic data, the real ionization path cannot be determined and the energies of all competing processes such as charge transfer, protonation, and hydride abstraction need to be compared.

## 1 Introduction

Atmospheric pressure chemical ionization (APCI) and electrospray ionization (ESI) are two of the most important interfaces used for LC-MS in analytical applications. While ESI has become near ubiquitous for the development of routine analytical methods with LC-MS, APCI remains important for analytes with low molecular weight and low polarity which are not ionized or show poor signal with ESI.^1−4^ The APCI ion source designs relevant for LC-MS use heating vaporizers and nebulizers to enable direct infusion of analyte solutions, and considerable efforts have been devoted to study the effect of the solvents on the ionization mechanism in APCI.^1−3,5^ Importantly for APCI, ionization of the solvents themselves leads to formation of new ion species that influence the ionization of the analytes. From this point of view, the solvents used in typical LC mobile phases may also be considered as potential modifiers, which is an important consideration in the development of new analytical methods with APCI.

Although toluene is one of the best-known dopants used in APPI,^6−8^ it is also used as a solvent (50–100% in methanol) for ionization of analytes in petroleum samples and environmentally relevant compounds such as polycyclic aromatic hydrocarbons (PAHs) with APCI. Addition of toluene improves the formation of M^+^ and increases the M^+^/[M + H]^+^ ratio, while in a mixture of toluene/methanol (50:50), dominant formation of [M + H]^+^ is observed.^9^ However, for practical LC-MS work, acetonitrile, methanol, dichloromethane, chloroform, and water are the most widely used solvents (i.e., mobile phase components), and many research groups have reported that these solvents influence the ionization efficiency and signal intensity with LC-MS equipped with APCI ion sources. For example, Marvin et al.^10^ reported that PAHs are ionized mainly through proton transfer in dichloromethane and acetonitrile solvents and that the signal intensity decreases as the fraction of dichloromethane increases. On the other hand, some authors have reported that acetonitrile suppresses the ionization signal and decreases formation of [M + H]^+^.^11−13^ Colizza et al.^11^ suggested that reduced formation of [M + H]^+^ in the presence of acetonitrile cannot be solely due to the thermodynamics of the proton transfer and the relative proton affinities of acetonitrile and the analyte, instead proposing that aggregation of the analyte and the solvent and formation of a neutral cluster may inhibit ionization.

**Scheme 1:**
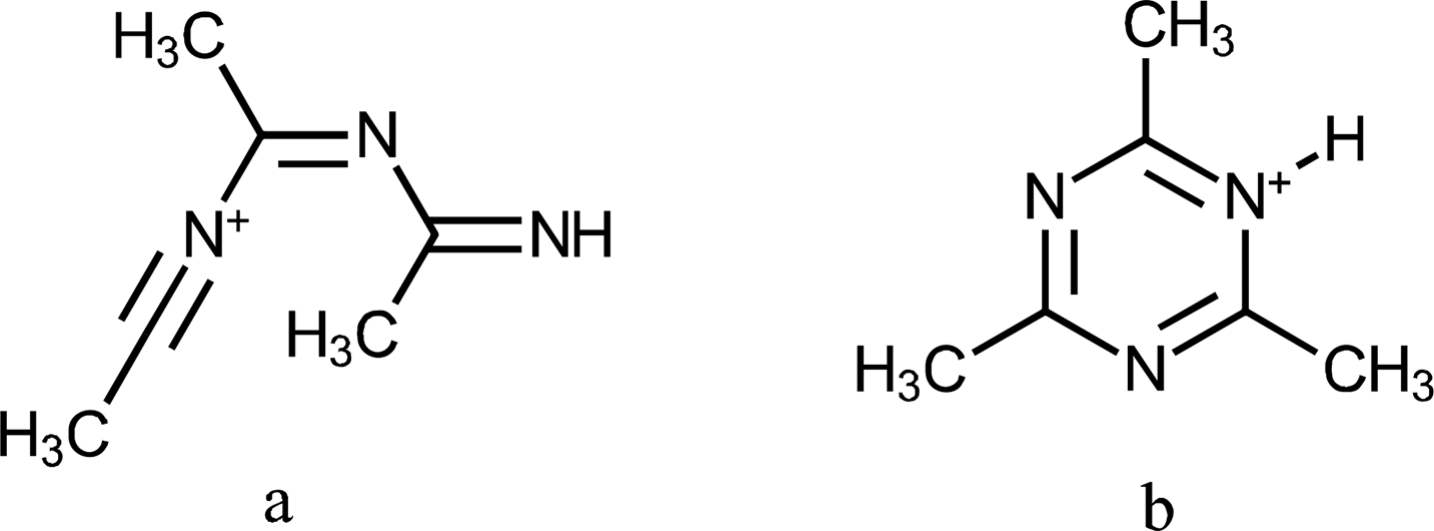
Proposed Structures for (CH_3_CN)_3_H^+^^14^

To explore the effect of the solvents on the ionization of analytes, ionization of the solvents themselves in APCI and their product ions have also been investigated. Kolakowski et al.^14^ studied the ionization of acetonitrile and dichloromethane in corona discharge-APCI. They did not observe any peak for ionization of dichloromethane but reported that acetonitrile can form CH_3_CNH^+^, (CH_3_CN)_2_H^+^, and (CH_3_CN)_3_H^+^. Because of the stability of the (CH_3_CN)_3_H^+^, they proposed that this ion is a trimer with covalent bonds (Scheme 1) rather than a loosely bound cluster with hydrogen bonds.

Carroll et al.^15^ also detected no MS peak for chloroform in the positive mode of APCI because of its high ionization energy and high reactivity of the produced ions toward impurities. However, Nicoletti et al.^16^ showed that ionization of chloroethanes produces chlorinated cations, [M – H]^+^ and [M – HCl]^+^ in an air atmosphere. It seems the ionization mechanisms are not the same in all APCI ion sources, and other than thermodynamic and structural properties of the analytes, additional parameters such as APCI design may influence the nature of the produced ions.

Because most of the analytes in APCI produce M^+^ and [M + H]^+^ in different ratios, charge transfer and protonation are considered as two competing processes controlled by IE and PA of the analytes.^17−20^ To take the effect of solvent into account, IE values of solvents have been also considered in previous studies. However, Herrera et al.^18^ showed that there is no correlation between IE values of the solvents CH_3_CN, CH_3_OH, and C_7_H_8_ and formation of the radical cation of the analyte, M^+^. Although direct ionization of the analyte in the discharge or charge transfer from N_2_^+^ to the analyte are two possible mechanisms of M^+^ formation, these pathways cannot be in full agreement with the experimental observations reported.^18^ Thus, the complexity of the APCI mechanism in the presence of the solvents arises from the reality that we do not know whether the solvents participate in the ionization or not; it may be that an analyte is ionized by an ion that is not stable enough to arrive at the inlet of MS to be detected for confirmation.^21^ To overcome this problem, Wolf et al.^22^ studied the effect of solvents and gases on ion formation in dielectric barrier discharge ionization using isotope labeling and showed that the proton of [M + H]^+^ originated from the solvent. Also, by using CO_2_ instead of air and N_2_, additional ions such as [M + OH]^+^ and [M – H]^+^ were detected indicating the role of CO_2_^+^ in the ionization of the analytes. Additionally, because the ion chemistry changes with distance from the discharge,^15^ the entrance of the analyte into the ion source or the geometry of the ionization region are also determining factors. The directions of the flowing gases in the ionization region may also carry the analyte into the discharge where the primary ions N_2_^+^, N_4_^+^, and O_2_^+^ are responsible for the ionization or take it away from the discharge where secondary ions such as (H_2_O)_*n*_H_3_O^+^ ionize the analyte via proton transfer.^15,23^

In the present work, we aim to interpret the complexity of the solvent effect on the APCI ionization based on competitive reactions via systematic experiments using relevant chemical standards with a standard instrumental setup on a commercial high-resolution MS platform. Ionization of nonpolar compounds with different structural and energetic properties are studied in acetonitrile, methanol, and chloroform. To determine the role of solvents in the ionization mechanism, volatile compounds that can be injected with and without solvent as vapor were also measured. To rationalize the experimental observations, density functional theory (DFT) and G4MP2 methods were used to compute energies of possible ionization paths and determine structures of the product ions.

## 2 Materials and Methods

### 2.1 Chemicals

Benzene (99.8%), toluene (99.7%), chlorobenzene (99.7%), benzonitrile (99%), butyrophenone (99%), 1-phenyl-2-butanone (98%), 4-phenyl-2-butanone (98%), retinol (95%), pseudoionone (>90%), 2,6-dimethyl-2,4,6-octatriene (80%), tetracene (98%), pentacene (99%), and chloroform (99.9%) were purchased from Sigma-Aldrich (Vienna, Austria). Benzo[*a*]pyrene (99.6%) was from LGC Standard. Methanol (99.9%) and acetonitrile (99.9%) were purchased from Honeywell. For the solid analytes (PAHs and retinol), solutions with concentrations of 50 μmol/L were prepared in pure solvents, while for the liquid analytes (benzene derivatives and ketones) the concentration of the measured solutions was 0.01% v/v. The head space of the volatile analytes (benzene derivatives and ketones) was injected into the ionization source as vapor samples. A syringe pump (KD Scientific, series 100, USA) was used to inject the solutions with flow rate of 20 μL min^–1^ into the nebulizer. A commercially available tune mix (ESI-L Low Concentration Tuning Mix, G1969-85000, Agilent Technologies) was prepared according to manufacturer instructions for tuning and accurate mass calibration of the mass spectrometer.

### 2.2 Instrumentation

An Agilent 6560 IM-QTOF mass spectrometer was used for all measurements. The instrument was calibrated prior to measurements in the 2 GHz extended dynamic range mode using the standard Dual Jetstream ESI ion source and following the recommended tune procedure of the manufacturer prior to changing to the corona discharge APCI source (G1947B, Agilent Technologies) for experimental measurements in positive mode. The temperature and flow rate of the drying gas were 200 °C and 13 L min^–1^, respectively. The temperature of the vaporizer and pressure of the nebulizer were 350 °C and 30 psi (207 kPa), respectively. The drift tube was operated with a pressure of 3.94 Torr (525.3 Pa) at 26–27.25 °C with high-purity nitrogen as the drift gas (Linde Gas GmbH, Vienna). A trap release time of 150 μs, trap filling time (10 ms), and maximum arrival time of 60 ms was applied for all IM-MS measurements. The operational parameters of the ion source are summarized in Table S1 (Supporting Information).

### 2.3 Data Processing

MassHunter Qualitative Analysis 10.0 and IM-MS Browser 10.0 (Agilent Technologies) were used for evaluation of the QTOFMS and IM-QTOFMS data, respectively. The measured accurate masses are tabulated in Tables S3 and S4 (Supporting Information).

### 2.4 Computational Methods

Density functional theory (DFT) with the B3LYP functional was used for the structural optimization of all neutral and charged molecules in the gas phase. The basis set 6-311++G(d,p) which includes both polarization and diffuse functions was used for all calculations. Frequency calculations were carried out at the same level of theory to compute thermodynamic properties including proton affinities (PA), gas phase basicities (GB), and Δ*H* and Δ*G* of the deprotonation reactions. Furthermore, the G4MP2 method was used for calculations of the ionization energies (IE) of the compounds for which an experimental IE was not available in the literature. All calculations were performed with Gaussian 16 software.^24^

## 3 Results and Discussion

### 3.1 Benzene and Its Derivatives

The first step in the study of the ionization mechanism is determination of the reactant ions (RIs) experimentally produced in the ion source. RIs such as N_2_^+^, N_4_^+^, and O_2_^+^ are very reactive and short-lived species that react with the molecules between the discharge needle and the MS inlet. Hence, these RIs cannot be detected with regular ion source geometries.^15,21^ The employed QTOFMS instrument cannot measure ions *m*/*z* of below 50 u with sub-1 ppm mass errors, which means that the detection and identity confirmation of other RIs such as NO^+^, H_3_O^+^, and NH_4_^+^ are not unambiguous. However, a peak with a *m*/*z* of 45.99 in the background spectrum corresponding to NO_2_^+^ (Figure S1) supports the existence of NO_*x*_^+^ ions in the ionization region (mass error 0.65 mDa). Furthermore, we tried to elucidate the potential role of RIs by monitoring the product ions of different analytes and calculation of their formation energies.

**Figure 1:**
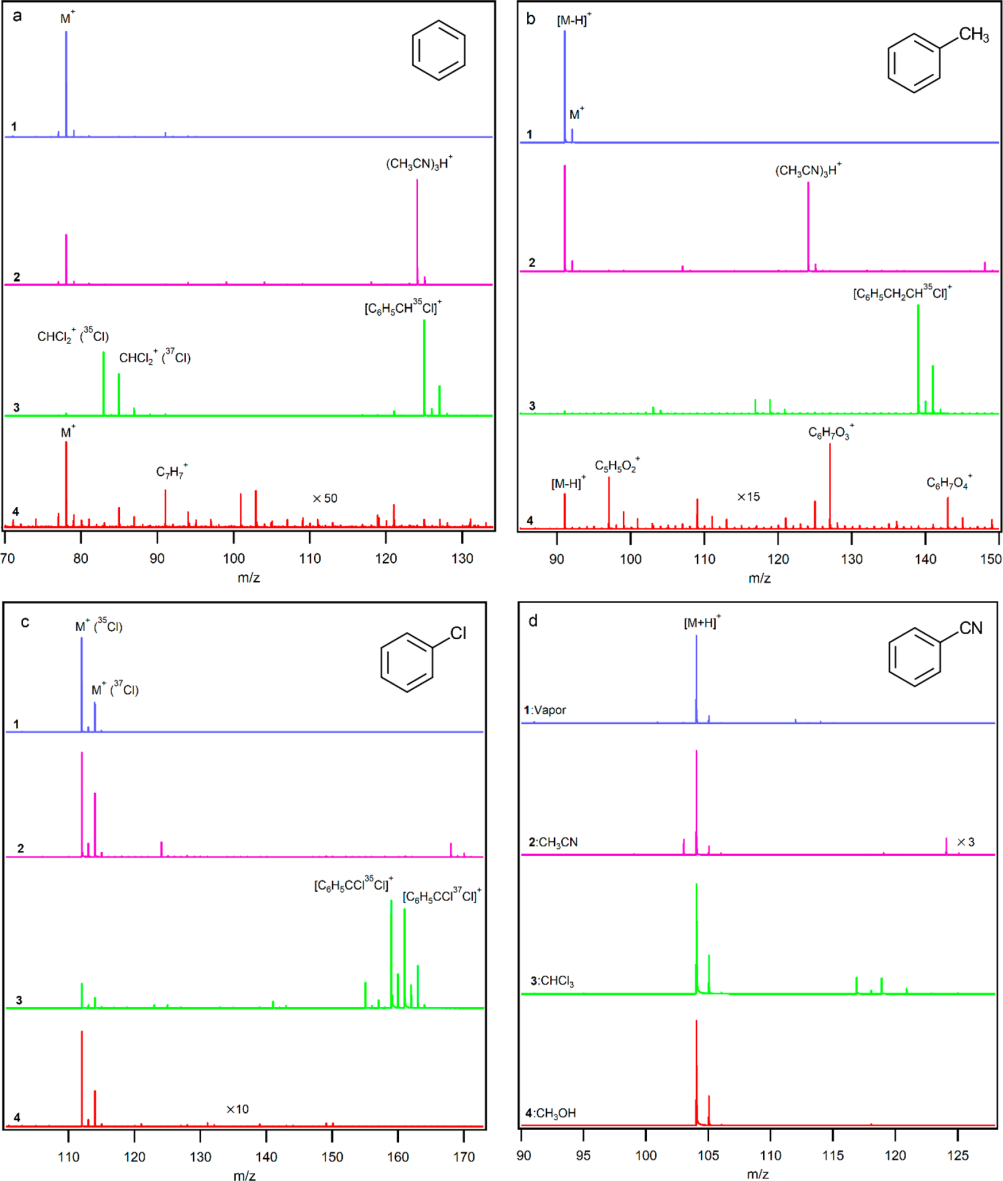
Mass spectra of (a) benzene, (b) toluene, (c) chlorobenzene, and (d) benzonitrile in (1) vapor and in solvents (2) acetonitrile, (3) chloroform, and (4) methanol (concentration: 0.01% v/v).

Figure 1 shows mass spectra of (a) benzene, (b) toluene, (c) chlorobenzene, and (d) benzonitrile infused as (1) vapors and diluted in (2) acetonitrile, (3) chloroform, and (4) methanol solvents. In the absence of the solvents, benzene, toluene, and chlorobenzene are ionized by charge transfer and hydride abstraction, indicating the presence of ions such as N_2_^+^, O_2_^+^, NO^+^, and NO_2_^+^, while protonation of benzonitrile confirms existence of H_3_O(H_2_O)_*n*_^+^. NH_4_^+^ may also be formed in the ion source, but because of the lower PA of benzonitrile compared to NH_3_ (Table 1), it cannot protonate benzonitrile. The solvents methanol, chloroform, and acetonitrile with IEs of 10.85, 11.37, and 12.20 eV, respectively, do not compete with the benzene derivatives in charge transfer reactions; however, they can influence protonation of these compounds. Mass spectra results show that benzene without any functional group is ionized as M^+^ in the absence of any solvent as well as in acetonitrile or methanol, while it produces a [M + CCl]^+^ ion in chloroform. The ionization energy (IE) of benzene is 9.24 eV (Table 1); hence, one (or more) ion(s) with an electron affinity (EA) of at least 9.24 eV is responsible for the ionization of benzene. The IEs of NO_2_, NO, O_2_, and N_2_ are 9.60, 9.26, 12.07, and 15.58 eV, respectively; hence, NO_2_^+^, NO^+^, O_2_^+^, and N_2_^+^ (Table 1) can ionize benzene via charge transfer. At the same time, these ions can ionize benzene by hydride abstraction. However, comparison of the charge transfer and hydride abstraction energies shows that the charge transfer is the more favorable path (Table 2) in accordance with the experimental mass spectra.

**Table 1:** Ionization Energies (IE), Proton Affinities (PA), and Gas Phase Basicity (GB) for the Studied Molecules in This Work

	IE (eV)	PA (kJ mol^–1^)		GB (kJ mol^–1^)	
molecule	exptl	exptl	B3LYP	exptl	B3LYP
benzene	9.24^a^	750.4^b^	765.9	725.4^b^	736.7
toluene	8.83^c^	784.0^b^	802.7	756.3^b^	772.6
chlorobenzene	9.08^d^	753.1^b^	769.1	724.6^b^	739.2
benzonitrile	9.73^e^	811.5^b^	826.7	780.9^b^	797.2
tetracene	6.97^f^	905.5^b^	929.3	876.5^b^	901.8
pentacene	6.61^f^		966.9		940.1
benzo[a]pyrene	7.12^g^		923.8		895.9
butyrophenone	9.1 ± 0.1^h^		881.6		851.5
1-phenyl-2-butanone	8.7^i^, 8.3^j^		866.6		852.8
4-phenyl-2-butanone	9.0 ± 0.1^k^		863.8		823.9
retinol	6.8 ± 0.2^l^		994.7^v^		1009.9^v^
pseudoionone	8.0^i^, 7.7^j^		938.6		906.5
2,6-dimethyl-2,4,6-octatriene	7.3^i^, 6.9^j^		944.2		920.0
methanol	10.85^m^	754.3^b^	753.6	724.5^b^	723.2
chloroform	11.37^n^		670.2		654.4
acetonitrile	12.20^o^	779.2^b^	785.7	748.0^b^	757.4
(CH_3_CN)_3_			918.2		885.4
H_2_O	12.65^p^	691.0^b^	687.8	660.0^b^	659.5
NH_3_	10.07^q^	853.6^b^	852.8	819.0^b^	824.5
N_2_	15.58^r^	493.8^b^	489.2	464.5^b^	450.1
O_2_	12.07^s^	421.0^b^	414.1	396.3^b^	389.3
NO	9.26^t^	531.8^b^	523.9	505.3^b^	497.2
NO_2_	9.60 ± 0.03^u^	591.0^b^	575.4	560.3^b^	544.5

a From ref (26).

b From ref (27).

c From ref (28).

d From ref (29).

e From ref (30).

f From ref (31).

g From ref (32).

h From ref (33).

i Calculated by G4MP2.

j Calculated by B3LYP/6-311++G(d,p).

k From ref (34).

l From ref (35).

m From ref (36).

n From ref (37).

o From ref (38).

p From ref (39).

q From ref (40).

r From ref (41).

s From ref (42).

t From ref (43).

u From ref (44).

v The PA and GB of retinol have been calculated for MOH + H^+^ → M^+^ + H_2_O.

**Table 2:** Comparison of the Energies of the Competing Ionization Pathways of Benzene Derivatives, Charge Transfer, and Hydride Abstraction by N_2_^+^, O_2_^+^, NO_2_^+^, and NO^+^^a^

charge transfer^b^	Δ*E*	hydride abstraction^c^	Δ*H*	Δ*G*
C_6_H_6_ + N_2_^+^ → C_6_H_6_^+^ + N_2_	–611.7	C_6_H_6_ + N_2_^+^ → C_6_H_5_^+^ + HN_2_	–269.6	–279.3
C_7_H_8_ + N_2_^+^ → C_7_H_8_^+^ + N_2_	–651.3	C_7_H_8_ + N_2_^+^ → C_7_H_7_^+^ + HN_2_	–472.4	–475.0
C_6_H_5_Cl + N_2_^+^ → C_6_H_5_Cl^+^ + N_2_	–627.2	C_6_H_5_Cl + N_2_^+^ → C_6_H_4_Cl^+^ + HN_2_	–242.7	–254.2
C_6_H_5_CN + N_2_^+^ → C_6_H_5_CN^+^ + N_2_	–564.4	C_6_H_5_CN + N_2_^+^ → C_6_H_4_CN^+^ + HN_2_	–203.4	–213.8
C_6_H_6_ + O_2_^+^ → C_6_H_6_^+^ + O_2_	–273.1	C_6_H_6_ + O_2_^+^ → C_6_H_5_^+^ + HO_2_	–164.7	–175.0
C_7_H_8_ + O_2_^+^ → C_7_H_8_^+^ + O_2_	–312.6	C_7_H_8_ + O_2_^+^ → C_7_H_7_^+^ + HO_2_	–367.5	–370.7
C_6_H_5_Cl + O_2_^+^ → C_6_H_5_Cl^+^ + O_2_	–288.5	C_6_H_5_Cl + O_2_^+^ → C_6_H_4_Cl^+^ + HO_2_	–137.8	–149.9
C_6_H_5_CN + O_2_^+^ → C_6_H_5_CN^+^ + O_2_	–225.8	C_6_H_5_CN + O_2_^+^ → C_6_H_4_CN^+^ + HO_2_	–98.5	–109.5
C_6_H_6_ + NO^+^ → C_6_H_6_^+^ + NO	–1.9	C_6_H_6_ + NO^+^ → C_6_H_5_^+^ + HNO	119.9	111.5
C_7_H_8_ + NO^+^ → C_7_H_8_^+^ + NO	–41.5	C_7_H_8_ + NO^+^ → C_7_H_7_^+^ + HNO	–82.9	–84.2
C_6_H_5_Cl + NO^+^ → C_6_H_5_Cl^+^ + NO	–17.4	C_6_H_5_Cl + NO^+^ → C_6_H_4_Cl^+^ + HNO	146.8	136.6
C_6_H_5_CN + NO^+^ → C_6_H_5_CN^+^ + NO	45.3	C_6_H_5_CN + NO^+^ → C_6_H_4_CN^+^ + HNO	186.1	177.0
C_6_H_6_ + NO_2_^+^ → C_6_H_6_^+^ + NO_2_	–34.7	C_6_H_6_ + NO_2_^+^ → C_6_H_5_^+^ + HNO_2_	–16.0	–36.7
C_7_H_8_ + NO_2_^+^ → C_7_H_8_^+^ + NO_2_	–74.3	C_7_H_8_ + NO_2_^+^ → C_7_H_7_^+^ + HNO_2_	–218.8	–232.4
C_6_H_5_Cl + NO_2_^+^ → C_6_H_5_Cl^+^ + NO_2_	–50.2	C_6_H_5_Cl + NO_2_^+^ → C_6_H_4_Cl^+^ + HNO_2_	10.9	–11.6
C_6_H_5_CN + NO_2_^+^ → C_6_H_5_CN^+^ + NO_2_	12.5	C_6_H_5_CN + NO_2_^+^ → C_6_H_4_CN^+^ + HNO_2_	50.2	28.8

a The energies are in kJ mol^–1^.

b Charge transfer energies were calculated using the experimental IEs in Table 1.

c Hydride abstraction energies (Δ*H* and Δ*G* values) were calculated by the B3LYP/6-311++G(d,p) method.

Toluene vapor as well as in acetonitrile or methanol is ionized via hydride abstraction and formation of an (M – H)^+^ ion. Furthermore, in methanol, some peaks were observed for C_5_H_5_O_2_^+^, C_6_H_7_O_3_^+^, and C_6_H_7_O_4_^+^ attributed to protonated forms of hydroxy-cyclopentadiene-one, trihydroxy benzene, and tetrahydroxy benzene. However, in chloroform, [M + CCl]^+^ is the main product observed. Interestingly, toluene with a lower IE (8.83 eV) than benzene produces only a small amount of M^+^ in vapor and in acetonitrile. Comparison of the charge transfer and hydride abstraction energies of toluene (Table 2) shows that, in the presence of O_2_^+^, NO_2_^+^, and NO^+^, hydride abstraction is more favored than charge transfer while charge transfer in the presence of N_2_^+^ is thermodynamically more favorable than hydride abstraction by N_2_^+^. As mass spectra results show that toluene is ionized mainly by hydride abstraction, it is concluded that N_2_^+^ cannot be responsible for the ionization of toluene or does not exist in appreciable quantities in the ionization region. These results also indicate that the ionization mechanism is based on competing reactions and confirms that the absolute values of IEs cannot be used directly for determination of the real ionization pathway.

Chlorobenzene, like benzene, produces M^+^ in vapor, in acetonitrile, and in methanol. However, unlike benzene, which produces only [M + CCl]^+^, it produces both [M + CCl]^+^ and M^+^ in chloroform, which is in agreement with lower IE of chlorobenzene (9.08 eV) compared to that of benzene (9.24 eV). Gas phase basicities (GB) of benzene and chlorobenzene (∼725 kJ mol^–1^) are lower than that of (CH_3_CN)_3_ (∼885 kJ mol^–1^); hence, these compounds are not protonated in acetonitrile. GBs of benzene and chlorobenzene are similar to that of methanol; hence, the solvent with higher concentration is protonated while benzene and chlorobenzene are preferably ionized via the more favored charge transfer pathway.

Benzonitrile, with the highest IE (9.73 eV) and PA (811.5 kJ mol^–1^) within the benzene derivatives studied, is mainly ionized via protonation and formation of [M + H]^+^. The Δ*H* value for protonation of benzonitrile by H_3_O^+^ is about −120 kJ mol^–1^. The electron transfer energies from benzonitrile to N_2_^+^, O_2_^+^, NO_2_^+^, and NO^+^ are −564.4, −225.8, 12.54, and 45.3 kJ mol^–1^, respectively. According to this data, in the presence of N_2_^+^ and O_2_^+^, we should observe a strong M^+^ peak for benzonitrile, but it is absent in the mass spectrum. Hence, it can be concluded that N_2_^+^ and O_2_^+^ probably have a minor role at most in the ionization region of this ion source. Because NO_2_^+^ and NO^+^ cannot theoretically ionize benzonitrile by charge transfer and given that the mass spectrum shows that benzonitrile does not form M^+^, it is postulated that NO_2_^+^ and NO^+^ may be two of the main RIs in this ion source and play more important roles than N_2_^+^ and O_2_^+^ in the ionization processes. In fact, the presence of NO^+^ in high abundance is not surprising. While corona discharge ion sources normally produce (H_2_O)_*n*_H^+^ as the major RI, small changes in the design and geometry of the ion source have been reported to lead to formation of NO^+^ as the main RI in atmospheric air.^25^

Comparison of the mass spectra recorded with and without solvents shows that, although chloroform alters the ionization mechanism, acetonitrile and methanol do not have such an influence. However, methanol is observed to reduce the extent of M^+^ and [M – H]^+^ formation while enhancing protonation, [M + H]^+^. This is because of the lower basicity of methanol which does not prevent the analytes from being protonated. In the case of benzonitrile and methanol, benzonitrile is the stronger base (PA of 811.5 kJ mol^–1^) and is more favorably protonated. Even if the methanol (PA = 754.3 kJ mol^–1^) is protonated, it will donate a proton to benzonitrile since it is a weaker base than benzonitrile. Hence, in the case of compounds with proton acceptor sites (where other ionization paths are not possible) methanol enhances the ionization via the protonation pathway. On the other hand, acetonitrile enhances M^+^ and [M – H]^+^ formation but decreases the extent of protonation. Acetonitrile has the highest IE (12.2 eV) of the solvents studied and cannot compete with benzene (9.24 eV) and chlorobenzene (9.08 eV) for ionization via M^+^ formation. Acetonitrile forms (CH_3_CN)_3_H^+^ as the only product ion in the ion source used in this study (Figure S2a). Formation of (CH_3_CN)_3_H^+^ is probably via a nucleophilic attack of N atom of a neutral CH_3_CN to the C atom of CH_3_CNH^+^ and ends with formation of (CH_3_CN)_3_H^+^ with a six-membered ring.^14^ In attempts to yield in-source fragmentation, an increase in the capillary fragmentor voltage did not lead to conversion of (CH_3_CN)_3_H^+^ into smaller clusters such as (CH_3_CN)_2_H^+^ and CH_3_CNH^+^ indicating that (CH_3_CN)_3_H^+^ is not a loosely bound cluster. Instead, it is probably a triazine derivative as previously proposed (Scheme 1b).^14^ (CH_3_CN)_3_ with a calculated PA and GB of about 918 and 885 kJ mol^–1^, respectively, is a strong base in the gas phase; hence, acetonitrile decreases the protonation of benzonitrile.

For the analytes observed to only undergo protonation, use of acetonitrile as the solvent led to a decrease in the signal intensity. We postulate that this competitive mechanism hypothesis can explain the problem with the signal suppression in acetonitrile reported by other researchers.^11−13^ We note here that different APCI geometries will influence the extent of this behavior. APCI sources that yield CH_3_CNH^+^ instead of (CH_3_CN)_3_H^+^ as the main product ion of acetonitrile will not yield such a strong decrease in the protonation pathway due to the lower PA of CH_3_CN (∼780 kJ mol^–1^) compared to (CH_3_CN)_3_.

**Scheme 2:**
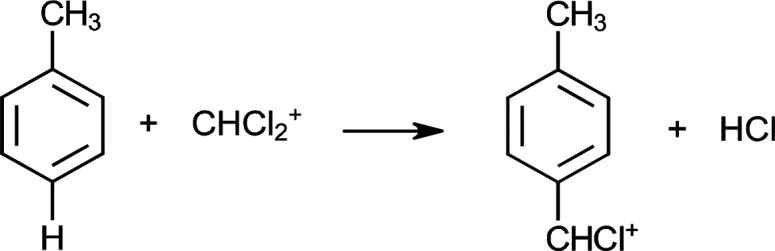
Ionization of Toluene in the Presence of CHCl_2_^+^ by Electrophilic Substitution

The mass spectrum of neat chloroform shows that CHCl_2_^+^ is the main product ion, but a small amount of CCl_3_^+^ is also formed (Figure S2b). It is suggested that formation of CHCl_2_^+^ is because of the protonation of CHCl_3_ followed by HCl elimination. The B3LYP-calculated Δ*H* and Δ*G* values for the reaction CHCl_3_ + H^+^ → CHCl_2_^+^ + HCl are −649 and −662 kJ mol^–1^, respectively, indicating the thermodynamic favorability of CHCl_2_^+^ formation via this mechanism. In the presence of CHCl_2_^+^, benzene, toluene, and chlorobenzene produce [M – H + CHCl]^+^ via electrophilic substitution followed by elimination of HCl. For benzene and chlorobenzene, substitution occurs at the carbons of the aromatic ring. Since toluene has both sp^2^ and sp^3^ hybridized carbon atoms, different possible isomers of [toluene – H + CHCl]^+^ were considered and their energies were compared (Figure S3). Comparison of the relative energies shows that the most favored path is substitution of the para hydrogen atom of toluene with CHCl_2_^+^ (Scheme 2).

Although the mass spectra show that CHCl_2_^+^ mainly ionizes the benzene derivatives by electrophilic substitution, its ability to ionize these compounds via charge transfer was also investigated theoretically. The G4MP2-calculated electron affinity of CHCl_2_^+^ is 8.3 eV. This indicates that CHCl_2_^+^ cannot ionize benzene, toluene, benzonitrile, and chlorobenzene via charge transfer, which is in agreement with the experimental results.

### 3.2 Polycyclic Aromatic Hydrocarbons (PAHs)

**Figure 2:**
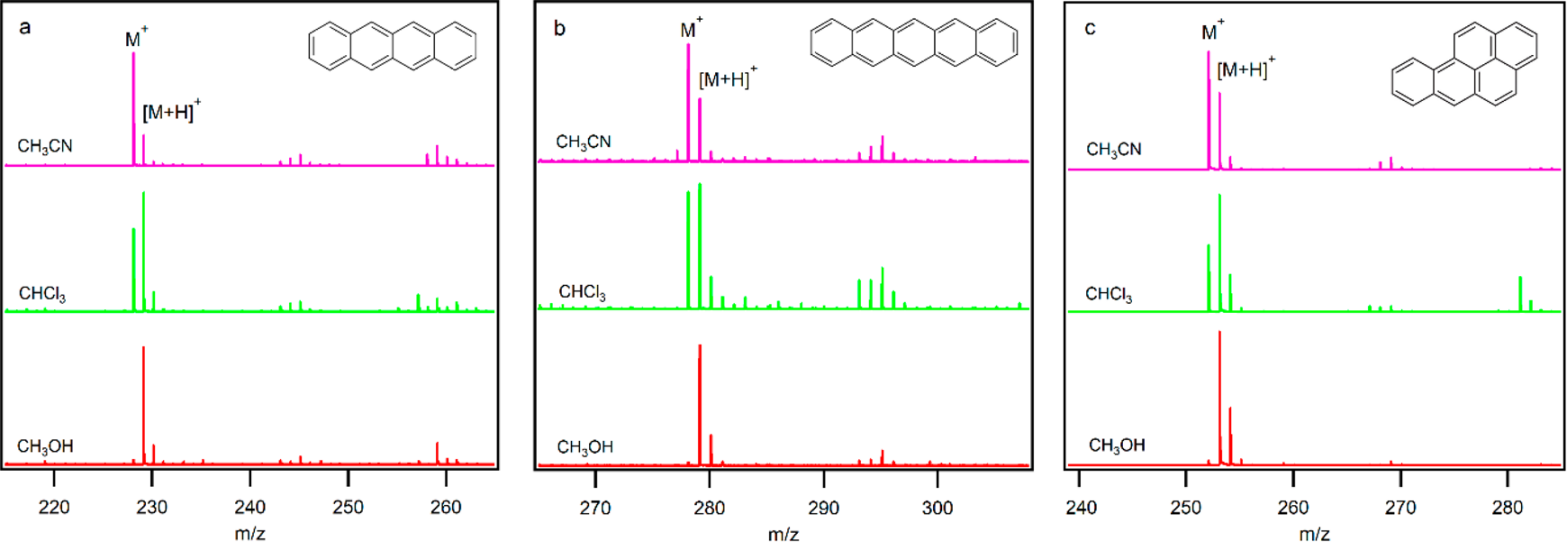
Mass spectra of (a) tetracene, (b) pentacene, and (c) benzo[*a*]pyrene in acetonitrile, chloroform, and methanol solvents (concentrations are 50 μmol/L).

Having elucidated some key characteristics of the ionization behavior of this ion source with small analytes with key structural differences, a broader range of analytes with more diverse properties were considered again with a focus on typical LC-MS solvents. Since the measurement of PAHs in different solvents using APCI is of broad analytical and environmental relevance,^6,8,10^ we first studied the effect of different solvents on the ionization mechanisms of tetracene, pentacene, and benzo[*a*]pyrene as representative analytes. Figure 2 shows the mass spectra of these PAHs in acetonitrile, chloroform, and methanol. Tetracene, pentacene, and benzo[*a*]pyrene with smaller IE values (6.97, 6.61., and 7.12 eV, respectively) than benzene have a higher tendency to be ionized via charge transfer. However, the PAHs also have higher basicities than benzene (Table 1); hence, their ionization mechanism is different to that of benzene and, other than charge transfer yielding M^+^, they are also protonated to [M + H]^+^ indicating the presence of H_3_O^+^ as a main RI. To determine the most basic proton acceptor site, protonation from all carbon atoms of tetracene, pentacene, and benzo[*a*]pyrene was studied theoretically (Figure S4). Comparison of the relative energies of the protonated isomers of the PAHs show that the central rings are more basic than the terminal rings. The M^+^/[M + H]^+^ ratio in the solvents decreased in the order acetonitrile > chloroform > methanol. This trend is in agreement with the discussion in the previous section. Methanol, with a PA of 754 kJ mol^–1^, is not a strong competitor of PAHs (PA = 923–967 kJ mol^–1^) for protonation, and [M + H]^+^ ions are observed as the main product ions of PAHs in the presence of methanol. In other words, when there is no strong basic solvent, protonation is more favored than charge transfer and hydride abstraction. Comparison of the data in Table 3 shows that, from the two competing pathways of charge transfer and hydride abstraction, the former is the more favorable ionization mechanism of the PAHs in the presence of N_2_^+^, O_2_^+^, NO_2_^+^, and NO^+^. Hence, hydride abstraction can be discarded and charge transfer and protonation energies should be compared. The Δ*H* values for protonation of the PAHs by H_3_O^+^ are −238, −276, and −233 kJ mol^–1^, respectively. The charge transfer energies in Table 3 show that the charge transfer from the PAHs to N_2_^+^, O_2_^+^, and NO_2_^+^ is more favorable than protonation of these compounds, which is not in agreement with the experimental mass spectra. In other words, if N_2_^+^, O_2_^+^, and NO_2_^+^ exist in the ionization region, a peak for M^+^ in methanol should be observed. Protonation is only more favorable than charge transfer in the presence of NO^+^, which is in agreement with the experimental data.

**Table 3:** Comparison of the Energies of the Competing Ionization Pathways of PAHs, Charge Transfer and Hydride Abstraction, by N_2_^+^, O_2_^+^, NO_2_^+^, and NO^+^^a^

charge transfer^b^	Δ*E*	hydride abstraction^c^	Δ*H*	Δ*G*
C_18_H_12_ + N_2_^+^ → C_18_H_12_^+^ + N_2_	–830.7	C_18_H_12_ + N_2_^+^ → C_18_H_11_^+^ + HN_2_	–359.6	–368.7
C_22_H_14_ + N_2_^+^ → C_22_H_14_^+^ + N_2_	–865.5	C_22_H_14_ + N_2_^+^ → C_22_H_13_^+^ + HN_2_	–404.3	–413.6
C_20_H_12_ + N_2_^+^ → C_20_H_12_^+^ + N_2_	–816.3	C_20_H_12_ + N_2_^+^ → C_20_H_11_^+^ + HN_2_	–362.7	–372.1
C_18_H_12_ + O_2_^+^ → C_18_H_12_^+^ + O_2_	–492.1	C_18_H_12_ + O_2_^+^ → C_18_H_11_^+^ + HO_2_	–254.7	–264.4
C_22_H_14_ + O_2_^+^ → C_22_H_14_^+^ + O_2_	–526.8	C_22_H_14_ + O_2_^+^ → C_22_H_13_^+^ + HO_2_	–299.4	–309.3
C_20_H_12_ + O_2_^+^ → C_20_H_12_^+^ + O_2_	–477.6	C_20_H_12_ + O_2_^+^ → C_20_H_11_^+^ + HO_2_	–257.8	–267.8
C_18_H_12_ + NO^+^ → C_18_H_12_^+^ + NO	–220.9	C_18_H_12_ + NO^+^ → C_18_H_11_^+^ + HNO	29.9	22.1
C_22_H_14_ + NO^+^ → C_22_H_14_^+^ + NO	–255.7	C_22_H_14_ + NO^+^ → C_22_H_13_^+^ + HNO	–14.8	–22.8
C_20_H_12_ + NO^+^ → C_20_H_12_^+^ + NO	–206.5	C_20_H_12_ + NO^+^ → C_20_H_11_^+^ + HNO	26.8	18.7
C_18_H_12_ + NO_2_^+^ → C_18_H_12_^+^ + NO_2_	–253.7	C_18_H_12_ + NO_2_^+^ → C_18_H_11_^+^ + HNO_2_	–106.0	–126.1
C_22_H_14_ + NO_2_^+^ → C_22_H_14_^+^ + NO_2_	–288.5	C_22_H_14_ + NO_2_^+^ → C_22_H_13_^+^ + HNO_2_	–150.7	–171
C_20_H_12_ + NO_2_^+^ → C_20_H_12_^+^ + NO_2_	–239.3	C_20_H_12_ + NO_2_^+^ → C_20_H_11_^+^ + HNO_2_	–109.1	–129.5

a The energies are in kJ mol^–1^.

b Charge transfer energies were calculated using the experimental IEs in Table 1.

c Hydride abstraction energies (Δ*H* and Δ*G* values) were calculated by the B3LYP/6-311++G(d,p) method.

In acetonitrile, because of the high PA of (CH_3_CN)_3_ (918 kJ mol^–1^), it competes with the PAHs for protonation. In other words, (CH_3_CN)_3_ depletes H_3_O^+^ in the ionization region and NO^+^ finds the opportunity to ionize PAHs by charge transfer and leads to formation of M. These experimental and theoretical data confirm that, other than H_3_O^+^ which is responsible for protonation of the PAHs, another possible RI is NO^+^. While CHCl_2_^+^ ionized the benzene derivatives by electrophilic substitution, the PAHs are observed to produce mainly M^+^ and [M + H]^+^ in chloroform because of their low IEs and high PAs.

### 3.3 Phenyl Butanone Isomers

**Figure 3:**
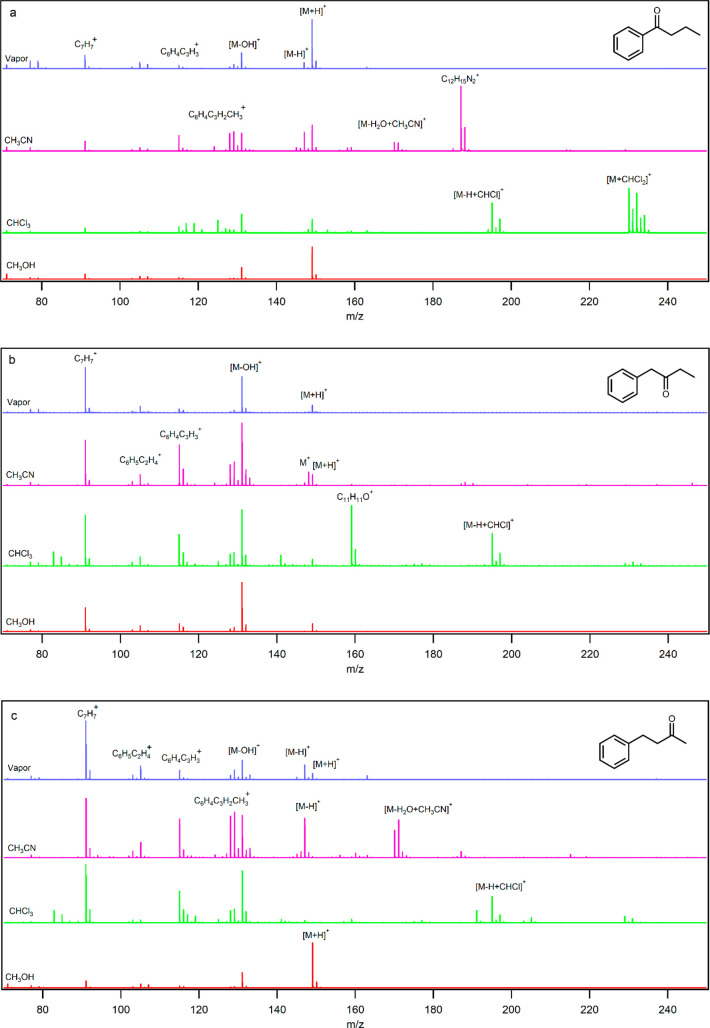
Mass spectra of (a) butyrophenone, (b) 1-phenyl-2-butanone, and (c) 4-phenyl-2-butanone in the gas phase and in acetonitrile, chloroform, and methanol solvents (concentrations of the phenyl butanone isomers in the solvent was 0.01% v/v).

Phenyl ketones with an aromatic ring, an alkyl group, and a C=O group as a proton acceptor site can be chemically ionized by charge transfer, hydride abstraction, and protonation, which makes them excellent model analytes for studying ionization mechanisms. It is well-known that, in ordinary APCI ion sources with (H_2_O)_*n*_H^+^ as RI, ketones are mainly ionized via protonation.^45−47^ However, the mass spectra of the vapor of phenyl butanone isomers measured using the present ion source show that these ketones are ionized by fragmentation, charge transfer (M^+^), protonation ([M + H]^+^), and hydride abstraction ([M – H]^+^) (Figure 3). The phenyl butanones (PA = 864–882 kJ mol^–1^) can be protonated by H_3_O^+^ and NH_4_^+^; however, as the PA of NH_3_ (854 kJ mol^–1^) is higher than that of H_2_O (691 kJ mol^–1^), protonation of these compounds by H_3_O^+^ is thermodynamically more favorable than NH_4_^+^. Furthermore, as no [M + NH_4_]^+^ ion was observed for the studied compounds, it can be concluded that abundance of NH_4_^+^ in the ion source is not high. Formation of M^+^ and [M – H]^+^ further supports the hypothesis that RIs other than H_3_O^+^ and NH_4_^+^ in the ionization mechanisms play an important role, in agreement with the discussion in the previous sections.

The B3LYP-optimized structures for possible isomers of [M – H]^+^ ions of the phenyl butanone isomers are provided in Figure S5. Although the mass spectra of these isomers appear complex, the results can be rationalized by the proposed ionization mechanisms and their respective structural and energetic properties. Table 4 compares the energies of the charge transfer and hydride abstraction of phenyl butanone isomers by N_2_^+^, O_2_^+^, NO^+^, and NO_2_^+^. In the presence of N_2_^+^ and O_2_^+^, charge transfer is more favorable than hydride abstraction which is not in agreement with the experimental data. Also, charge transfer from butyrophenone, 1-phenyl-2-butanone, and 4-phenyl-2-butanone to NO_2_^+^ (−287, −196, −343 kJ mol^–1^, respectively) is more favorable than their protonation by H_3_O^+^ (−191, −176, −171 kJ mol^–1^, respectively). Hence, if NO_2_^+^ exists as a main RI, it would suppress formation of [M + H]^+^ ions and only [M – H]^+^ ions and their fragments would be observed in the mass spectra. As the energies for hydride abstraction of phenyl butanones by NO^+^ and their protonation by H_3_O^+^ are comparable and the mass spectra show both the [M – H]^+^ and [M + H]^−^ ions, it can be concluded that NO^+^ and H_3_O^+^ are the main reactant ions in this ion source. For 1-phenyl-2-butanone, the charge transfer and hydride abstraction energies by NO^+^ are comparable; hence, M^+^ of this isomer is also observed, while only a weak signal is observed for the [M – H]^+^ ion of this isomer, probably because this ion is not stable and undergoes fragmentation. Other than reactant ions, solvent also influences the ionization processes mainly by competition for protonation. For example, butyrophenone is observed to be ionized mainly via protonation in vapor and in methanol, whereby there is no strong base to compete with it for protonation. However, in acetonitrile, because of the higher basicity of (CH_3_CN)_3_, protonation of the ketones is reduced and instead formation of M^+^ and [M – H]^+^ are favored.

**Table 4:** Comparison of the Charge Transfer and Hydride Abstraction Energies for the Phenyl Butanone Isomers, In the Presence of N_2_^+^, O_2_^+^, NO_2_^+^, and NO^+^^a^

charge transfer^b^	Δ*E*	hydride abstraction^c^	Δ*H*	Δ*G*
BP + N_2_^+^ → BP^+^ + N_2_	–625.2	BP + N_2_^+^ → [BP-H]^+^ + HN_2_	–540.7	–543.4
1P2B + N_2_^+^ → 1P2B^+^ + N_2_	–663.8	1P2B + N_2_^+^ → [1P2B–H]^+^ + HN_2_	–449.5	–471.9
4P2B + N_2_^+^ → 4P2B^+^ + N_2_	–634.9	4P2B + N_2_^+^ → [4P2B–H]^+^ + HN_2_	–596.2	–596.4
BP + O_2_^+^ → BP^+^ + O_2_	–286.5	BP + O_2_^+^ → [BP-H]^+^ + HO_2_	–435.8	–439.1
1P2B + O_2_^+^ → 1P2B^+^ + O_2_	–325.2	1P2B + O_2_^+^ → [1P2B–H]^+^ + HO_2_	–344.6	–367.6
4P2B + O_2_^+^ → 4P2B^+^ + O_2_	–296.2	4P2B + O_2_^+^ → [4P2B–H]^+^ + HO_2_	–491.3	–492.1
BP + NO^+^ → BP^+^ + NO	–15.4	BP + NO^+^ → [BP-H]^+^ + HNO	–151.2	–152.6
1P2B + NO^+^ → 1P2B^+^ + NO	–54.0	1P2B + NO^+^ → [1P2B–H]^+^ + HNO	–60.0	–81.1
4P2B + NO^+^ → 4P2B^+^ + NO	–25.1	4P2B + NO^+^ → [4P2B–H]^+^ + HNO	–206.7	–205.6
BP + NO_2_^+^ → BP^+^ + NO_2_	–48.2	BP + NO_2_^+^ → [BP-H]^+^ + HNO_2_	–287.1	–300.8
1P2B + NO_2_^+^ → 1P2B^+^ + NO_2_	–86.8	1P2B + NO_2_^+^ → [1P2B–H]^+^ + HNO_2_	–195.9	–229.3
4P2B + NO_2_^+^ → 4P2B ^+^ + NO_2_	–57.9	4P2B + NO_2_^+^ → [4P2B–H]^+^ + HNO_2_	–342.6	–353.8

a BP, butyrophenone; 1P2B, 1-phenyl-2-butanone; 4P2B, 4-phenyl-2-butanone. The energies are in kJ mol^–1^.

b Charge transfer energies were calculated using the experimental IEs in Table 1.

c Hydride abstraction energies (Δ*H* and Δ*G* values) were calculated by the B3LYP/6-311++G(d,p) method.

**Figure 4:**
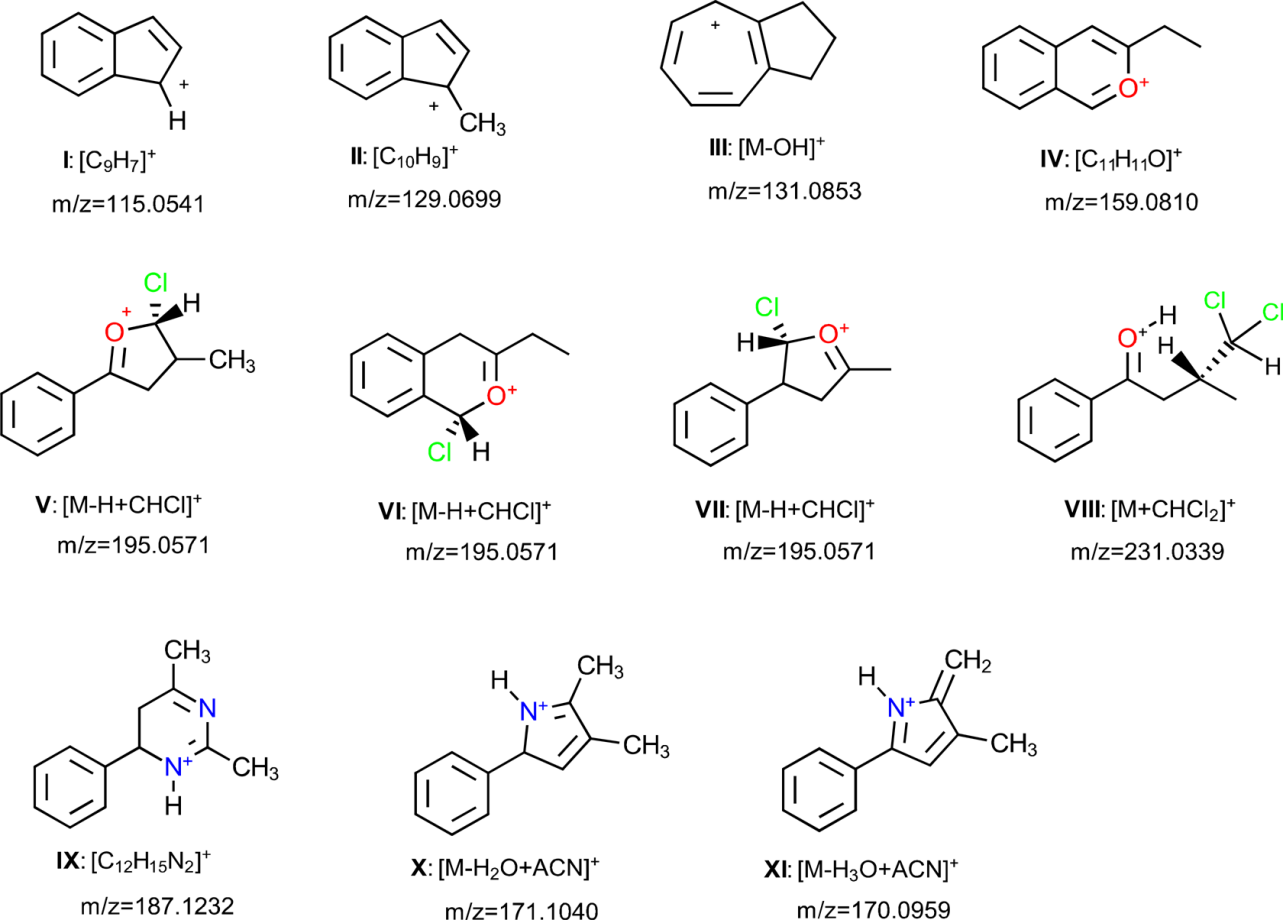
Structures of the most stable isomers of the fragments and ions observed in mass spectra of butyrophenone, 1-phenyl-2-butanone, and 4-phenyl-2-butanone.

Fragmentation patterns (peaks with *m*/*z* < 148) for the phenyl butanone isomers are broadly similar. C_7_H_7_^+^ is one of the major fragments of 1-phenyl-2-butanone and 4-phenyl-2-butanone, while its intensity in the butyrophenone mass spectrum is comparatively low. Other main fragments observed are C_6_H_4_C_3_H_3_^+^, C_6_H_4_C_4_H_5_^+^, and [M – OH]^+^ (structures **I**–**III** in Figure 4). On the other hand, the adduct ions of the solvents with the phenyl butanones are not the same. Formation of these ions is mainly controlled by the structures of the phenyl butanones rather than their energetic properties. The mass spectrum of butyrophenone in acetonitrile shows a peak for C_12_H_15_N_2_^+^ with a *m*/*z* of 187.1232, while this ion is not formed for the other phenyl butanones. Optimized structures for some possible isomers of C_12_H_15_N_2_^+^ are shown in Figure S6. The most stable isomer of C_12_H_15_N_2_^+^ has a dihydropyrimidine ring (structure **IX** in Figure 4). 4-Phenyl-2-butanone reacts with acetonitrile only after water elimination to produce [M – H_2_O + ACN]^+^ and [M – H_3_O + ACN]^+^. Comparison of the relative energies of possible isomers of these ions (Figure S7) shows that the most stable structures for these ions are those with a pyrrole ring (structures **X** and **XI** in Figure 4). 1-Phenyl-2-butanone shows an intense peak for [M – OH]^+^, and it does not form any ion with acetonitrile. It seems that this is because of the extraordinary stability of its [M – OH]^+^ ion with low tendency to react with the solvent. Comparison of the optimized structures of possible isomers of [M – OH]^+^ with molecular formula of C_10_H_11_^+^ in Figure S8 shows that the stability of [M – OH]^+^ ion of 1-phenyl-2-butanone may be due to formation of a cycloheptatrienyl (tropylium) cation (structure **III** in Figure 4).

In chloroform, butyrophenone produces some ions with *m*/*z* of 230–233 that were not observed for other phenyl butanones. The pair of peaks of 230.0496 and 232.0458 may be C_8_H_15_Cl_2_O_3_^+^ or C_11_H_13_Cl_2_N, while peaks 231.0339 and 233.0313 are due to [M + CHCl_2_]^+^. The optimized structures of [M + CHCl_2_]^+^ isomers for the phenyl butanones are shown in Figures S9–S11. Comparison of the relative stabilities of these isomers shows that the [M + CHCl_2_]^+^ ion of butyrophenone is more stable than for other phenyl ketones by about 50 kJ mol^–1^ (Figure S11). However, all the phenyl butanone isomers produced [M – H + CHCl]^+^ in chloroform. The optimized structures of the [M – H + CHCl]^+^ ions of the phenyl butanones (Figure S12 and Table S2) show that formation of this ion is thermodynamically favored for all phenyl butanones. An additional peak is observed for 1-phenyl-2-butanone in chloroform with *m*/*z* of 159.0810 and possible molecular formula of C_11_H_11_O^+^. This ion is probably an isochromenium derivative (structure **IV**) formed by electrophilic substitution of CHCl_2_^+^ at the ortho position of 1-phenyl-2-butanone followed by elimination of two HCl molecules.

In an APCI source with (H_2_O)_*n*_H^+^ as the RI, the ionization mechanism is based on proton transfer and it is expected that [M + H]^+^ is formed as the major product ion for the phenyl butanone isomers. Recently, Weller et al.^48^ studied ionization of monoterpenes in the presence of NO^+^ and reported that NO^+^ can ionize these compounds via hydride abstraction, charge transfer, and fragmentation. In our study, the mass spectra of the phenyl butanones in vapor with M^+^, [M – H]^+^, and many fragment ions also indicate the presence of NO^+^ as one of the main RIs. Furthermore, while ionization and fragmentation can also be due to diffusion of the neutral molecules into the discharge and reactions with electrons, N_2_^+^, and O_2_^+^, comparison of the thermodynamic data and mass spectra suggested that the probability of this pathway is low. The multichannel ionization mechanism of the phenyl butanones with several product ions also leads to complicated total ion mobility spectra with different patterns in different solvents (Figure S13).

Finally, in the case of LC-MS analysis, the properties of the employed mobile phase and lower concentrations of analytes studied must be considered for practical relevance. In the case of the mobile phase, a binary mixture of water and organic solvent such as acetonitrile is mandatory for elution from the LC column; hence, ionization of butyrophenone in binary mixtures of acetonitrile–water (90:10) and methanol–water (90:10) was studied (Figure S14). Addition of water to acetonitrile was observed to enhance formation of [M + H]^+^ because of the lower PA of H_2_O (691 kJ mol^–1^)^27^ compared to (CH_3_CN)_3_. The effects of water and methanol on the ionization mechanism of butyrophenone are observed to be similar, and the protonation is the main ionization mechanism in pure methanol and in the binary mixture of methanol and water. The effect of concentration (5–500 μmol/L) of butyrophenone on the ionization mechanism in acetonitrile and methanol was also studied. It was found that an increase in the concentration does not change the ionization mechanism and the produced ions, only the intensities of the observed signals (Figure S15).

### 3.4 Polyene Compounds

**Figure 5:**
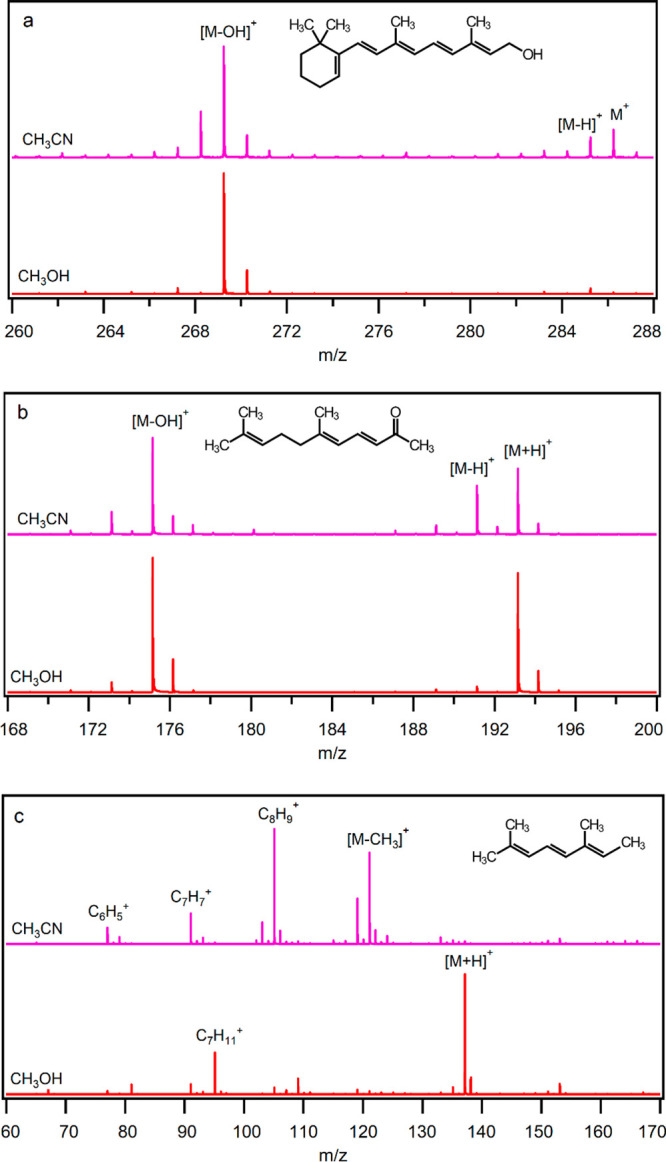
Mass spectra of (a) retinol, (b) pseudoionone, and (c) 2,6-dimethyl-2,4,6-octatriene in acetonitrile and methanol solvents.

Because of the existence of a conjugated π-electron system, polyenes can be ionized by loss of an electron to form M^+^.^49,50^ Furthermore, C(sp^3^)-H groups connected to the conjugated π-bonds assist ionization via hydride abstraction. Hence, ionization of polyenes proceeds through competitive reactions which can be influenced in the presence of different solvents. Retinol, 2,6-dimethyl-2,4,6-octatriene, and pseudoionone are unsaturated compounds with five, three, and two conjugated double bonds, respectively, while pseudoionone has a total of three double bounds. OH and C=O functional groups of retinol and pseudoionone, respectively, can act as proton acceptor sites. Figure 5 shows the mass spectra containing the major peaks for (a) retinol, (b) pseudoionone, and (c) 2,6-dimethyl-2,4,6-octatriene in acetonitrile and methanol. Retinol and pseudoionone also undergo fragmentation (Figure S16). No peak was observed corresponding to a protonated form of retinol in acetonitrile and methanol, presumably because of water elimination after protonation and formation of the stable cation [M – OH]^+^. The optimized structure of the protonated retinol shows that the [M + H]^+^ ion of retinol is not stable and it loses a water molecule to form a stable carbocation with charge delocalization on the conjugated π-system (Figure S17). In methanol, M^+^ and [M – H]^+^ ions are not observed. The calculated Δ*H* and Δ*G* values for protonation of retinol by H_3_O^+^ followed by water elimination are −304 and −350 kJ mol^–1^, respectively, while the calculated Δ*H* and Δ*G* values of hydride abstraction from retinol by NO^+^ are −336 and −341 kJ mol^–1^ (Table 5). The larger Δ*G* value of protonation is because of the entropic effect of water elimination which causes protonation to be thermodynamically more favorable than hydride abstraction by about 10 kJ mol^–1^. However, in acetonitrile, small peaks for M^+^ and [M – H]^+^ of retinol are observed which shows the importance of the solvent effect and competitive reactions; the ionization pathway is shifted from fragmentation (protonation followed by water elimination) to hydride abstraction and charge transfer leading to the observation of the precursor ion of retinol, M^+^. In acetonitrile, (CH_3_CN)_3_ with PA of 918 kJ mol^–1^ competes with retinol for protonation and allows other ionization paths leading to the formation of M^+^ and [M – H]^+^ species.

**Table 5:** Comparison of the Charge Transfer and Hydride Abstraction Energies for Retinol (C_20_H_30_O), Pseudoionone (C_13_H_20_O), and 2,6-Dimethyl-2,4,6-octatriene (C_10_H_16_) in the Presence of N_2_^+^, O_2_^+^, NO_2_^+^, and NO^+^^a^

charge transfer^b^	Δ*E*	hydride abstraction^c^	Δ*H*	Δ*G*
C_20_H_30_O + N_2_^+^ → C_20_H_30_O^+^ + N_2_	–847.2	C_20_H_30_O + N_2_^+^ → C_20_H_29_O^+^ + HN_2_	–725.3	–731.8
C_13_H_20_O + N_2_^+^ → C_13_H_20_O^+^ + N_2_	–731.4	C_13_H_20_O + N_2_^+^ → C_13_H_19_O^+^ + HN_2_	–573.3	–579.8
C_10_H_16_ + N_2_^+^ → C_10_H_16_^+^ + N_2_	–798.9	C_10_H_16_ + N_2_^+^ → C_10_H_15_^+^ + HN_2_	–619.0	–629.0
C_20_H_30_O + O_2_^+^ → C_20_H_30_O^+^ + O_2_	–508.5	C_20_H_30_O + O_2_^+^ → C_20_H_29_O^+^ + HO_2_	–620.4	–627.5
C_13_H_20_O + O_2_^+^ → C_13_H_20_O^+^ + O_2_	–392.7	C_13_H_20_O + O_2_^+^ → C_13_H_19_O^+^ + HO_2_	–468.4	–475.5
C_10_H_16_ + O_2_^+^ → C_10_H_16_^+^ + O_2_	–460.2	C_10_H_16_ + O_2_^+^ → C_10_H_15_^+^ + HO_2_	–514.1	–524.7
C_20_H_30_O + NO^+^ → C_20_H_30_O^+^ + NO	–237.4	C_20_H_30_O + NO^+^ → C_20_H_29_O^+^ + HNO	–335.8	–341.0
C_13_H_20_O + NO^+^ → C_13_H_20_O^+^ + NO	–121.6	C_13_H_20_O + NO^+^ → C_13_H_19_O^+^ + HNO	–183.8	–189.0
C_10_H_16_ + NO^+^ → C_10_H_16_^+^ + NO	–189.1	C_10_H_16_ + NO^+^ → C_10_H_15_^+^ + HNO	–229.5	–238.2
C_20_H_30_O + NO_2_^+^ → C_20_H_30_O^+^ + NO_2_	–270.2	C_20_H_30_O + NO_2_^+^ → C_20_H_29_O^+^ + HNO_2_	–471.7	–489.2
C_13_H_20_O + NO_2_^+^ → C_13_H_20_O^+^ + NO_2_	–154.4	C_13_H_20_O + NO_2_^+^ → C_13_H_19_O^+^ + HNO_2_	–319.7	–337.2
C_10_H_16_ + NO_2_^+^ → C_10_H_16_^+^ + NO_2_	–221.9	C_10_H_16_ + NO_2_^+^ → C_10_H_15_^+^ + HNO_2_	–365.4	–386.4

a The energies are in kJ mol^–1^.

b The charge transfer energies were calculated using the experimental IEs in Table 1.

c Hydride abstraction energies (Δ*H* and Δ*G* values) were calculated by the B3LYP/6-311++G(d,p) method.

Pseudoionone with the calculated PA of 938 kJ mol^–1^ is a stronger base than (CH_3_CN)_3_. Hence, in both methanol and acetonitrile, an intense peak for [M + H]^+^ is observed. Unlike retinol, protonated forms of pseudoionone are stable (Figure S18). The calculated energies for ionization of pseudoionone by NO^+^ via charge transfer and hydride abstraction are about −122 and −184 kJ mol^–1^, respectively, indicating that the hydride abstraction is more favored, in agreement with the [M – H]^+^ peak in acetonitrile.

Interestingly, 2,6-dimethyl-2,4,6-octatriene without any ordinary basic site is mainly ionized by protonation and forms [M + H]^+^ in methanol. Comparison of the optimized structures of the protonated forms of 2,6-dimethyl-2,4,6-octatriene shows that protonation of one of its C(sp^2^) atoms leads to stabilization of the [M + H]^+^ cation by both charge delocalization on the conjugated π-system and formation of a tertiary carbocation (Figure S19). The calculated PA for 2,6-dimethyl-2,4,6-octatriene is 944 kJ mol^–1^ indicting that it is a strong carbon base. In acetonitrile, protonation is suppressed and fragmentation (demethylation) becomes the main path of ionization of 2,6-dimethyl-2,4,6-octatriene. Hence, in acetonitrile, C_9_H_13_^+^ and C_8_H_9_^+^ related to the first and second demethylation are the major ions observed. In the case of the polyenes, not only do solvents change their ionization pathways, but also selection of a suitable solvent can reduce fragmentation. For retinol, acetonitrile decreased protonation to prevent water elimination after protonation, while methanol prevented octatriene from fragmentation by increasing the protonation path. Recently, ionization of carotenoids as large polyene compounds has been investigated in APCI in a mixture of methanol, water, and *tert*-butyl methyl ethers.^51^ The major signal in the mass spectra of these compounds was due to [M + H]^+^ ion indicating that methanol and water increase formation of protonated species.

## 4 Conclusion

This work can be divided into two main parts: the identification of the main RIs in a commercial APCI ion source and the effect of solvents acetonitrile, methanol, and chloroform on the ionization mechanisms for different classes of compounds. Since (H_2_O)_*n*_H^+^ mainly ionizes the analytes by protonation, ionization via charge transfer and hydride abstraction was attributed to other RIs such as NO^+^, NO_2_^+^, O_2_^+^, and N_2_^+^. Comparison of the calculated energies of all possible ionization pathways and the product ions showed that short-lived ions such as N_2_^+^ and O_2_^+^ cannot be responsible for the ionization of compounds; hence, NO^+^ along with (H_2_O)_*n*_H^+^ are identified as the main RIs in this ion source.

Other than RIs, structural and thermodynamic properties of the analytes influence the preferred ionization mechanism in this ion source. However, solvents can also compete with the analyte for ionization or produce new RIs and thereby change the preferred ionization mechanism. For example, PAHs with low IE and high PA can be ionized via both charge transfer and protonation, while acetonitrile with higher PA than methanol can reduce the protonation pathway as they are more easily protonated in methanol. Similarly, the presence of acetonitrile diminishes the protonation of polyenes and allows ionization via hydride abstraction. Ions produced directly from the ionization of solvents can also act as new RIs to ionize the analyte and change the ionization mechanism. This was observed to be most important in the case of chloroform which produces CHCl_2_^+^ allowing ionization of benzene derivatives via electrophilic substitution. Overall, these characteristics render this ion source as versatile for analysis of a wider range of compounds including those that are not ionized with ordinary CD-APCI ion sources. This can be advantageous for specialized analytical LC-MS and GC-APCI-MS applications in which the signal intensity for detection of selected species (i.e., analytical sensitivity) is often more important than the chemical nature of product ions.

Finally, comparison of the product ions and energies of the ionization reactions showed that, although a given ionization path is thermodynamically possible, it can be suppressed by a more favorable ionization pathway. Hence, by using only the absolute thermodynamic data of a compound including PA and IE, we cannot determine the exact ionization mechanism occurring in APCI and other paths should be considered and compared to experimental data to achieve comprehensive understanding.

## Supporting Information Available

Operational parameters of the IM-QTOF and APCI ion source (Table S1); background mass spectrum (Figure S1); APCI-mass spectra of pure solvents (Figure S2); structures of different isomers of [toluene – H + CHCl]^+^ (Figure S3); protonated forms of PAHs (Figure S4); optimized structures of hydride abstracted phenyl ketones (Figure S5); relative stabilities of different isomers of C_12_H_15_N_2_^+^ (Figure S6); relative stabilities of different isomers of [M – H_2_O + ACN]^+^ and [M – H_3_O + ACN]^+^ (Figure S7); relative stabilities of different isomers of [M – OH]^+^ for phenyl ketones (Figure S8); relative stabilities of different isomers of C_11_H_13_Cl_2_O^+^ (BuPh + CHCl_2_)^+^ (Figure S9); comparison of relative stabilities of different isomers of C_11_H_13_Cl_2_O^+^ (Figure S10); relative stabilities of [M + CHCl_2_]^+^ of phenyl ketones (Figure S11); optimized structures of different isomers of [M – H + CHCl]^+^ for phenyl ketones (Figure S12); calculated Δ*H* and Δ*G* of electrophilic substitution of CHCl_2_^+^ into phenyl ketones (Table S2); ion mobility spectra of phenyl ketones in different solvents (Figure S13); mass and ion mobility spectra of butyrophenone in mixture of solvents (Figure 14); mass spectra for different concentrations of butyrophenone (Figure 15); mass spectra of retinol and pseudoionone in acetonitrile and methanol (Figure S16); optimized structures of isomers of retinol (R), [R + H]^+^, (R – OH)^+^, and [R – H]^+^ (Figure S17); optimized structures of isomers of pseudoionone (PD), (PD + H)^+^ and (PD – H)^+^ (Figure S18); different isomers for [M + H]^+^ and [M – H]^+^ ions of 2,6-dimethyl-2,4,6-octatriene (Figure S19); measured and theoretical masses for the studied benzene derivatives and polycyclic aromatic hydrocarbons (Table S3); and measured and theoretical masses for the studied phenyl butanones and polyenes (Table S4) (PDF)
