# Supplementary material for: Significance of Competitive Reactions in an Atmospheric Pressure Chemical Ionization Ion Source: Effect of Solvent
Source: J Am Soc Mass Spectrom. 2022 May 12;33(6):961–73. doi: 10.1021/jasms.2c00034 (PMC9164235; doi:10.1021/jasms.2c00034)
Supplement: Supplementary file 1 — js2c00034_si_001.pdf [file js2c00034_si_001.pdf]

<u>Supporting Information for</u>: On the Significance of Competitive Reactions in an Atmospheric Pressure Chemical Ionization Ion Source: Effect of Solvent

Younes Valadbeigi*, Tim Causon*

University of Natural Resources and Life Sciences, Vienna, Department of Chemistry, Institute of Analytical Chemistry, Muthgasse 18, 1190 Vienna, Austria

---

* Corresponding authors

E-Mails: y.valadbeigi@boku.ac.at; tim.causon.boku.ac.at

University of Natural Resources and Life Sciences, Vienna

Department of Chemistry

Institute of Analytical Chemistry

Muthgasse 18, Vienna, 1190

Austria

Contents **Page**

Table S1. Operational parameters of the IM-QTOF and APCI ion source S3

Figure S1. Mass spectrum of the background of the ion source. S3

Figure S2. APCI-mass spectra of pure solvents acetonitrile, chloroform, and methanol S4

Figure S3. Comparison of different isomers of [Toluene-H+CHCl]$^+$ S5

Figure S4. Protonated forms of tetracene, pentacene, and benzo[a]pyrene S6

Figure S5. Optimized structures of hydride abstracted phenyl ketones S7

Figure S6. Relative stabilities of different isomers of $C_{12}H_{15}N_2^+$ S8

Figure S7. Relative stabilities of different isomers of [M-H_2O+ACN]$^+$ and [M-H_3O+ACN]$^+$ S9

Figure S8. Relative stabilities of different isomers of [M-OH]$^+$ for phenyl ketones S10

Figure S9. Relative stabilities of different isomers of $C_{11}H_{13}Cl_2O^+$ (BuPh+$CHCl_2$)$^+$ S11

Figure S10. Comparison of relative stabilities of different isomers of $C_{11}H_{13}Cl_2O^+$ S12

Figure S11. Relative stabilities of [M+$CHCl_2$]$^+$ of phenyl ketones S13

Figure S12. Optimized structures of different isomers of [M-H+CHCl]$^+$ for phenyl ketones S14

Table S2. Calculated ΔH and ΔG of electrophilic substitution of $CHCl_2^+$ into phenyl ketones S14

Figure S13. Ion mobility spectra of phenyl ketones in different solvents S15

Figure S14. Mass and ion mobility spectra of butyrophenone in mixture of solvents S16

Figure S15. Mass spectra for different concentrations of butyrophenone S17

Figure S16. Mass spectra of retinol and pseudoionone in acetonitrile and methanol S18

Figure S17. Optimized structures of isomers of retinol (R), [R+H]$^+$, (R-OH)$^+$, and [R-H]$^+$ S19

Figure S18. Optimized structures of isomers of pseudoionone (PD), PD.H$^+$, and (PD-H)$^+$ S20

Figure S19. Optimized structures of different isomers for [M+H]$^+$ and [M-H]$^+$ ions of 2,6-dimethyl-2,4,6-octatriene S21

Table S3. The measured and theoretical masses for the studied benzene derivatives and polycyclic aromatic hydrocarbons S22

Table S4. The measured and theoretical masses for the studied phenyl butanones and polyenes S23

Table S1. Operational parameters of the IM-QTOF and APCI ion source used in this work.

Parameter	value
Operational mode	Positive
Corona current	4 μA
Corona voltage	3500 V
Drying gas flow	13 L min^{-1}
Drying gas temperature	200 ^{o}C
Nebulizer pressure	30 psi (207 kPa)
Vaporizer Temperature	350 ^{o}C
Sample infusion flow rate	20 μl min^{-1}
Ion trap release time	150 μs
Ion trap filling time	10 ms
Drift electric field	17.22 V cm^{-1}
Drift tube pressure	3.94 Torr

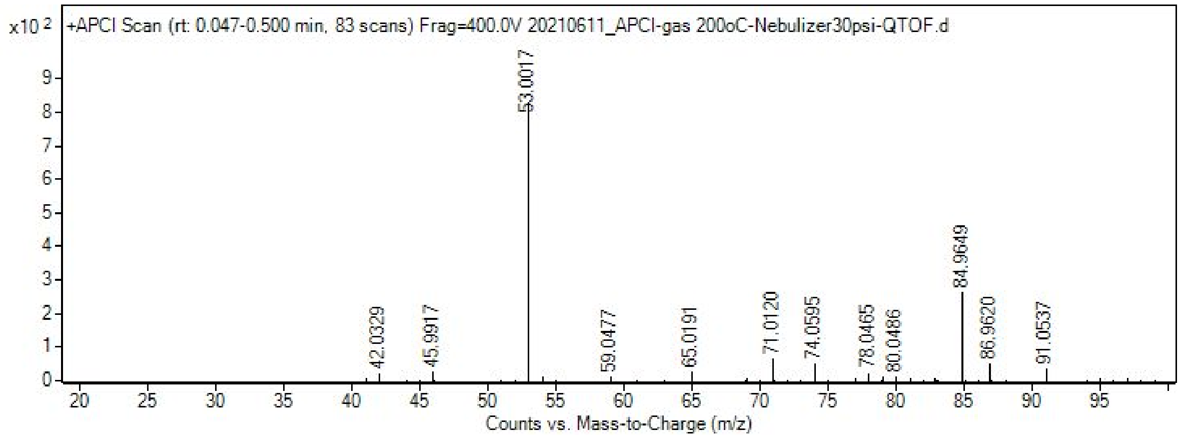

Figure S1. Mass spectrum of the background of the ion source. The peaks with *m/z* 84.96 and 86.96 are $(CH_2Cl_2)H^+$ may be due to CH_2Cl_2 or $CHCl_3$ contaminants. The peak with *m/z* of 91.054 can be attributed to $(H_2O)_4H_3O^+$ or $C_7H_7^+$, but because other smaller water clusters are not seen, it is probably $C_7H_7^+$, which is a main fragment of many organic compounds. The peak with *m/z* 42.0329 is protonated acetonitrile. The peak with *m/z* of 53.0017 may be due to C_3OH^+, $(CN)_2H^+$, and C_3H_3N , or $NH_4^+(NH_3)(H_2O)$. The small peak with *m/z* of 45.99 is NO_2^+.

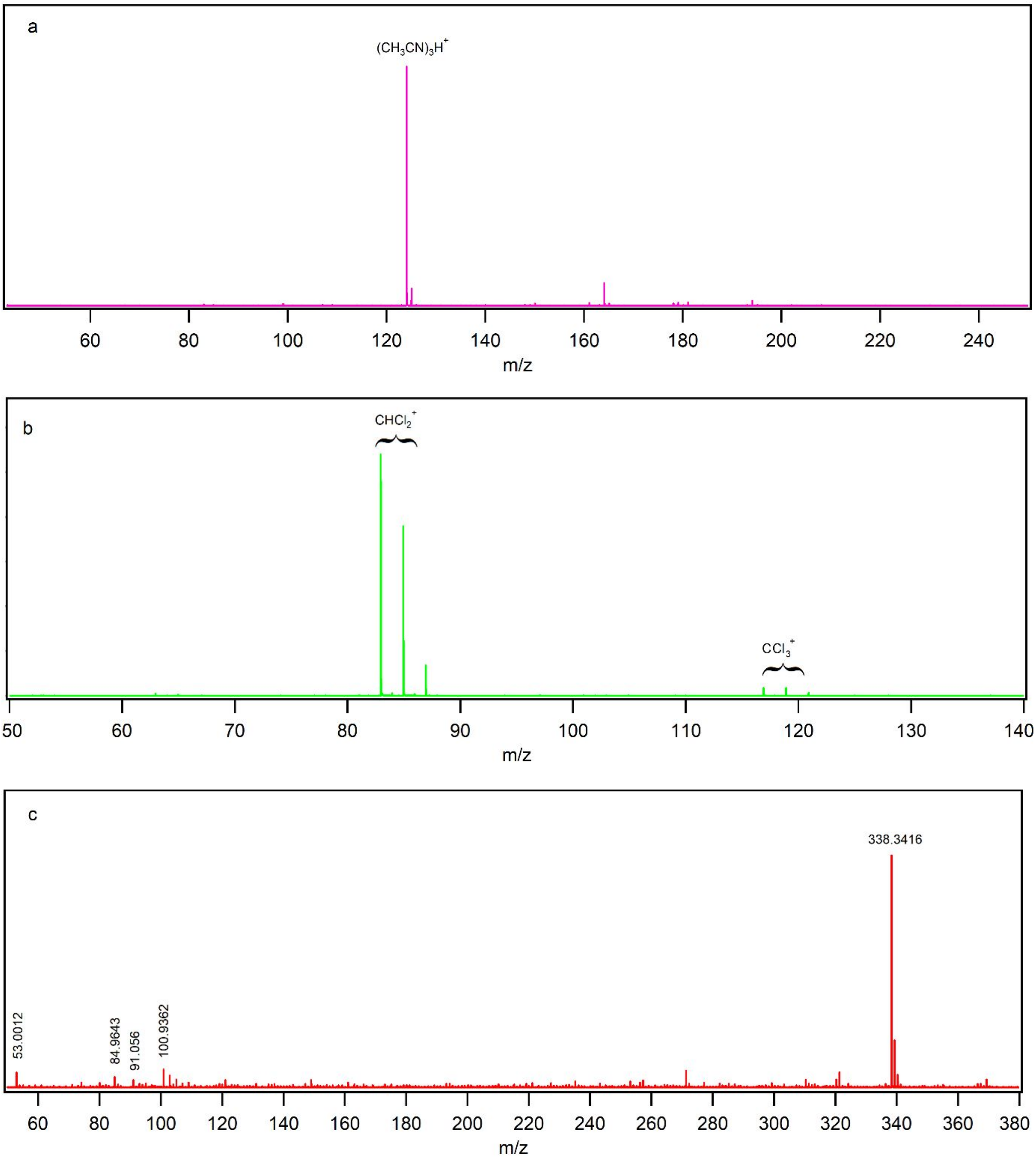

Figure S2. APCI-MS spectra of pure solvents (a) acetonitrile, (b) chloroform, and (c) methanol. The major peak for methanol is due to an ion with m/z=338.3416 which can be attributed to $[C_{22}H_{43}NO+H]^+$ or $[C_{20}H_{41}N_4+H]^+$ or an impurity in methanol.

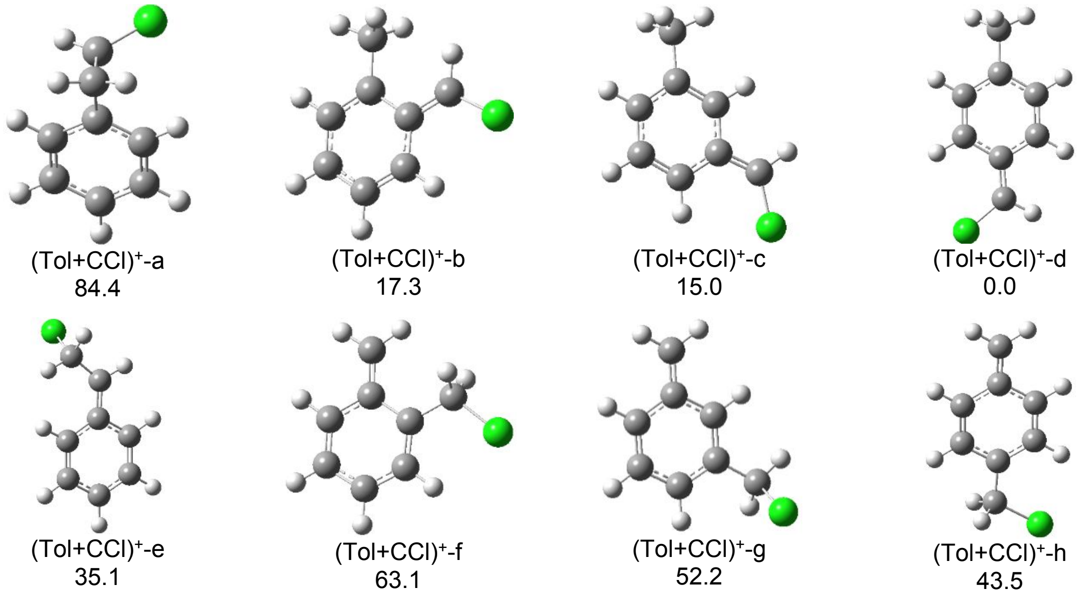

Figure S3. Comparison of relative energies for different possible isomers of [toluene-H+CHCl]$^+$. The energies are in kJ mol^{-1}.

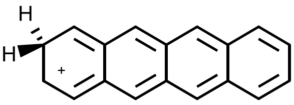
Ta
64.4

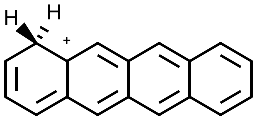
Tb
51.6

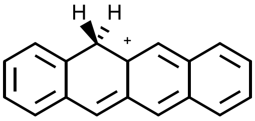
Tc
0.0

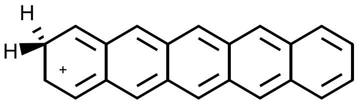
Pa
82.3

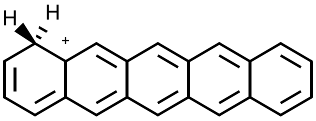
Pb
71.2

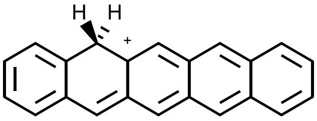
Pc
13.9

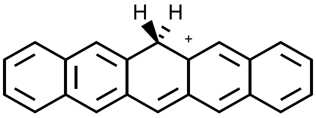
Pd
0.0

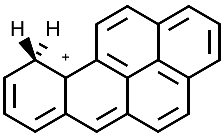
Ba
70.9

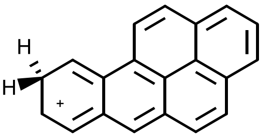
Bb
45.6

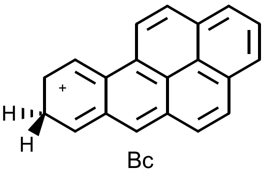
Bc
81.0

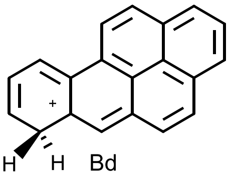
Bd
43.8

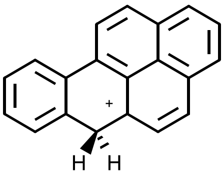
Be
0.0

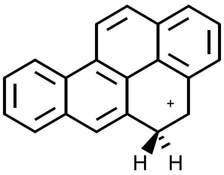
Bf
69.1

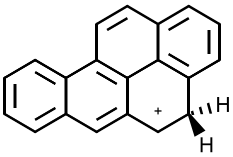
Bg
64.7

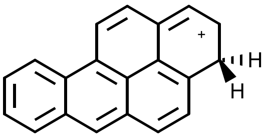
Bh
20.2

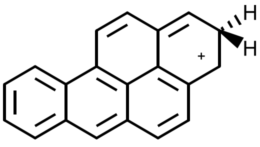
Bi
87.8

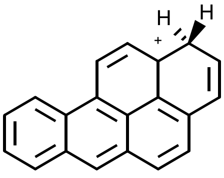
Bj
16.3

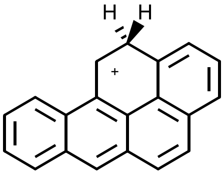
Bk
49.8

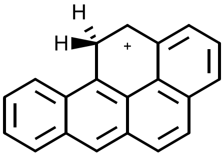
Bl
77.8

Figure S4. Relative energies of the protonated forms of tetracene (T), pentacene (P), and benzo[a]pyrene (B). The energies are in kJ mol^{-1}.

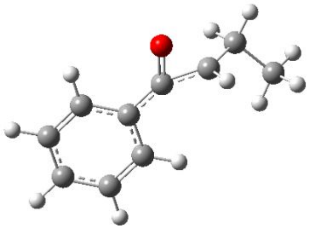

(BuPh-H)$^+$-a
96.7

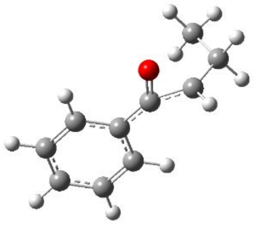

(BuPh-H)$^+$-b
96.0

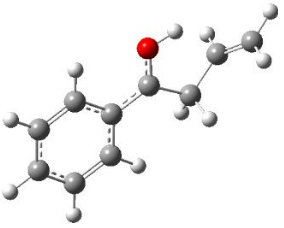

(BuPh-H)$^+$-c
0.0

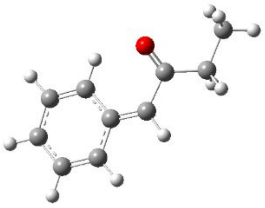

(1Ph-2Bu-H)$^+$-a
17.9

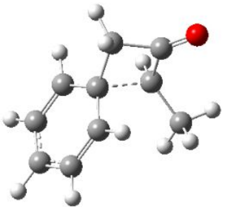

(1Ph-2Bu-H)$^+$-b
86.5

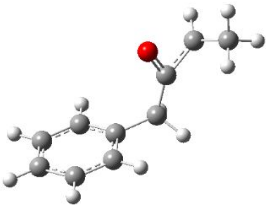

(1Ph-2Bu-H)$^+$-c
103.2

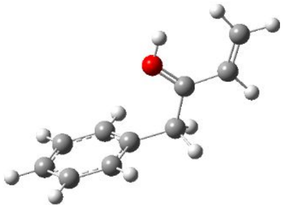

(1Ph-2Bu-H)$^+$-d
0.0

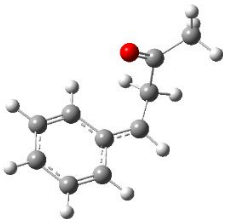

(4Ph-2Bu-H)$^+$-a
72.2

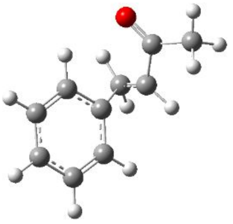

(4Ph-2Bu-H)$^+$-b
132.5

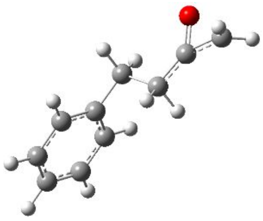

(4Ph-2Bu-H)$^+$-c
257.2

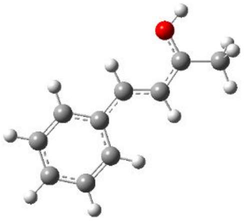

(4Ph-2Bu-H)$^+$-d
0.0

Figure S5. The optimized structures of different isomers of hydride abstracted butyrophenone (BuPh), 1-phenyl-2-butanone (1Ph-2Bu), and 4-phenyl-2-butanone (4Ph-2Bu). The relative energies are in kJ mol^{-1}.

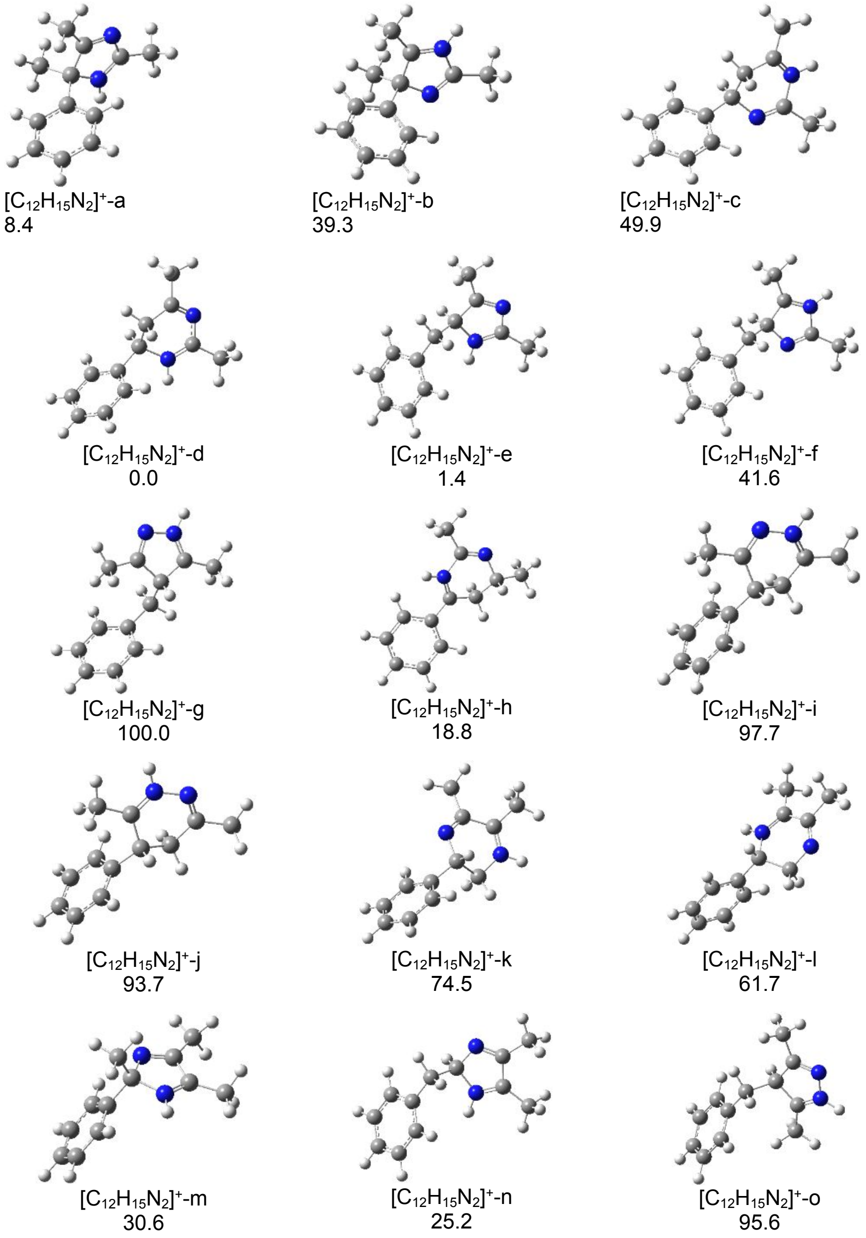

Figure S6. Comparison of relative stabilities of different isomers of $C_{12}H_{15}N_2^+$ with *m/z* of 187.1232.

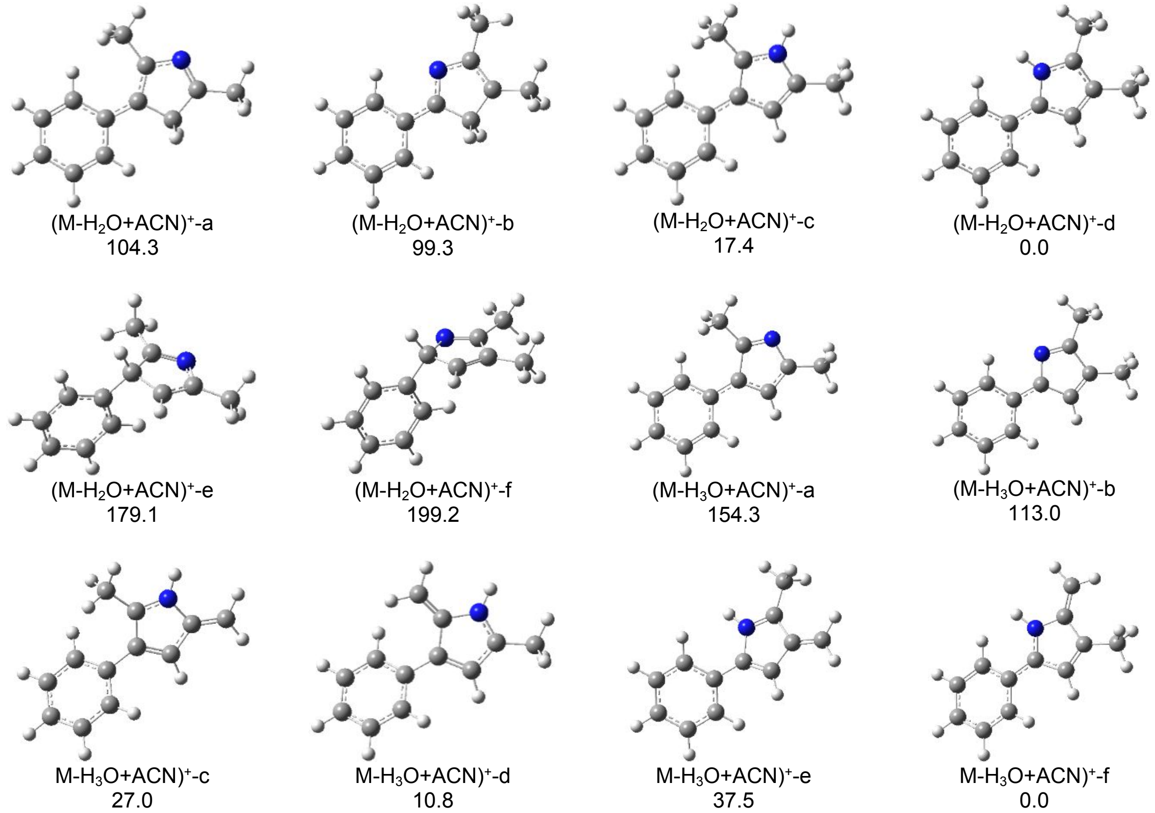

Figure S7. Comparison of relative stabilities of different isomers of [M-H_2O+ACN]$^+$ and [M-H_3O+ACN]$^+$ of 4-phenyl-2-butanone.

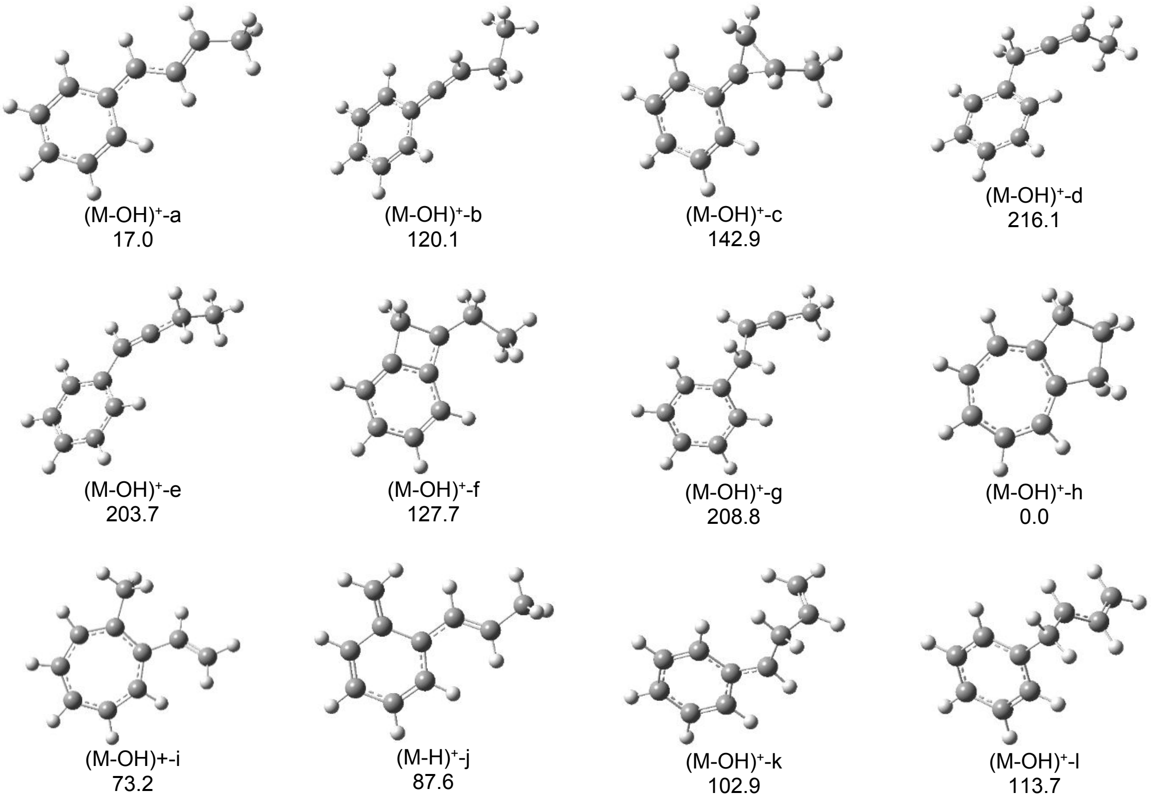

Figure S8. Comparison of relative stabilities of different isomers of $[M\text{-}OH]^+$ for butyrophenone, 1-phenyl-2-butanone, and 4-phenyl-2-butanone. The relative energies are in kJ mol^{-1}.

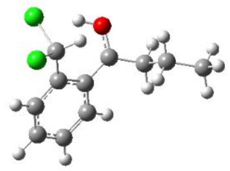
(BuPh+CHCl$_2$)$^+$-a
31.3

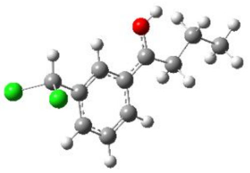
(BuPh+CHCl$_2$)$^+$-b
8.4

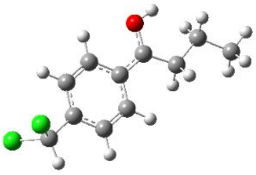
(BuPh+CHCl$_2$)$^+$-c
5.7

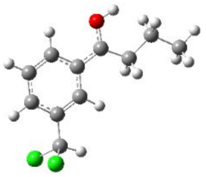
(BuPh-CHCl$_2$)$^+$-d
9.4

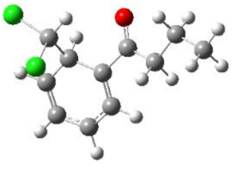
(BuPh+CHCl$_2$)$^+$-e
158.9

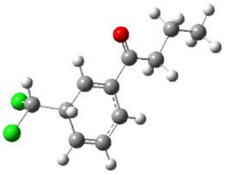
(BuPh+CHCl$_2$)$^+$-f
163.8

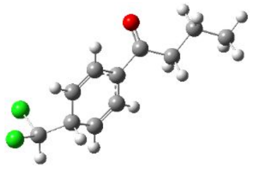
(BuPh+CHCl$_2$)$^+$-g
165.5

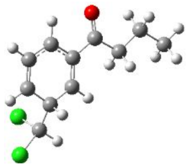
(BuPh-CHCl$_2$)$^+$-h
162.4

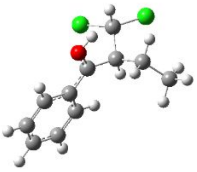
(BuPh-CHCl$_2$)$^+$-i
39.4

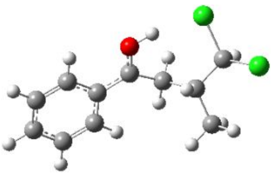
(BuPh+CHCl$_2$)$^+$-j
0.0

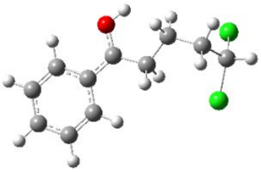
(BuPh+CHCl$_2$)$^+$-k
0.2

Figure S9. Comparison of relative stabilities of different isomers of $C_{11}H_{13}Cl_2O^+$ (BuPh+CHCl$_2$)$^+$ with *m/z* of 231.0339 observed for butyrophenone with chloroform solvent. The relative energies are in kJ mol^{-1}.

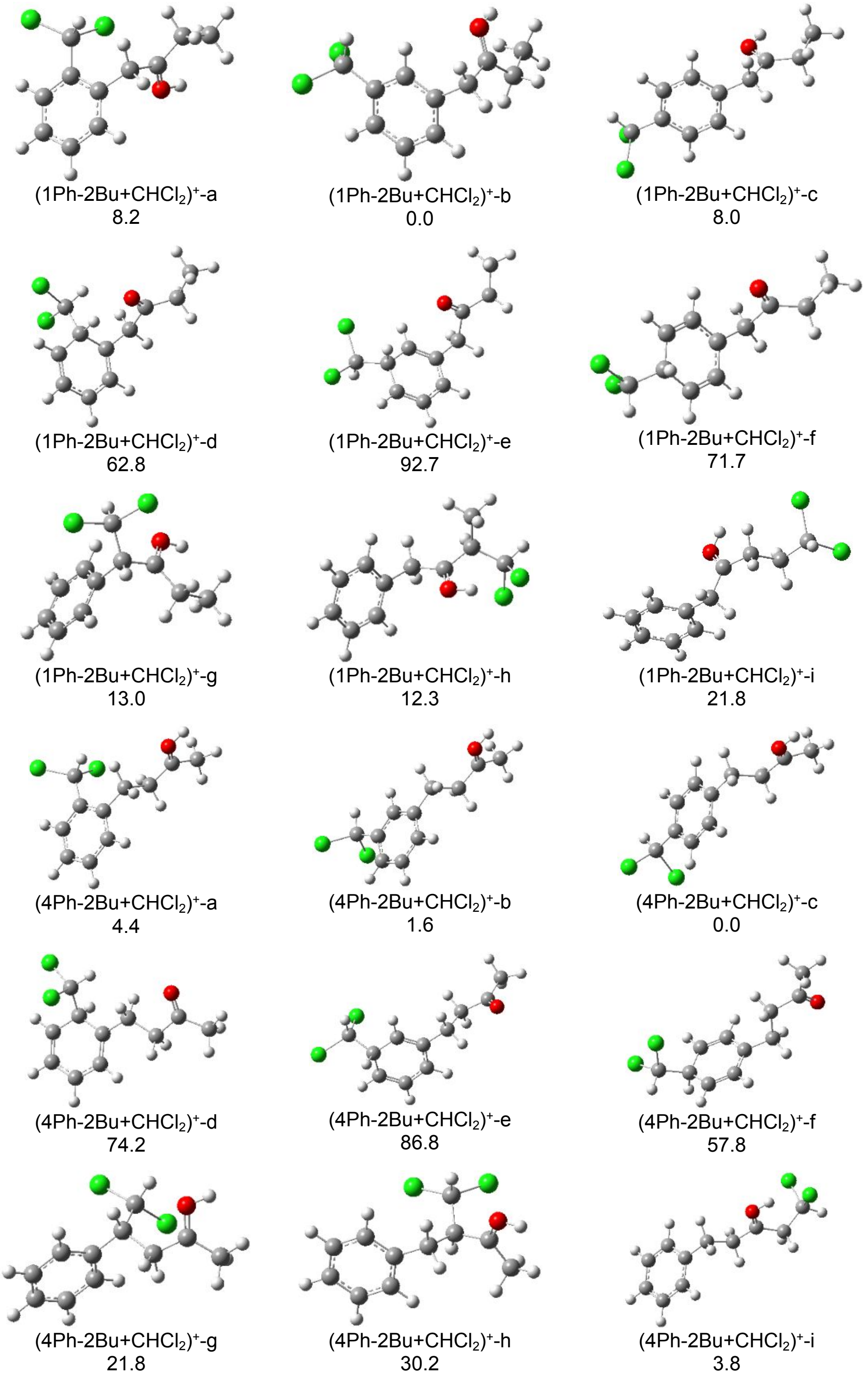

Figure S10. Comparison of relative stabilities of different isomers of $C_{11}H_{13}Cl_2O^+$ for 1-phenyl-2-butanone (1Ph-2Bu), and 4-phenyl-2-butanone (4Ph-2Bu). The relative energies are in kJ mol^{-1}. These ions were not observed in the mass spectra, but they were studied theoretically for comparison with the corresponding ions of butyrophenone which were observed in the MS spectrum.

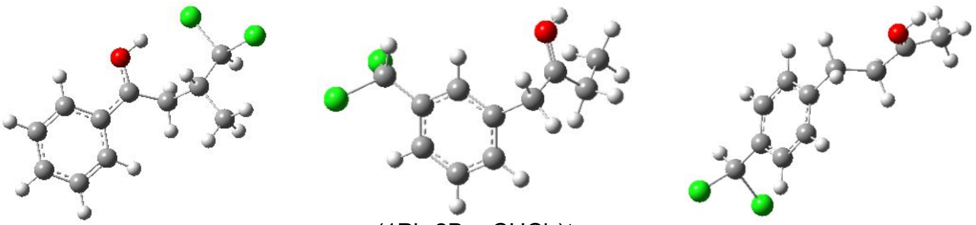

(BuPh+CCl_2)$^+$ 0.0 | (1Ph-2Bu+$CHCl_2$)$^+$ 41.3 | (4Ph-2Bu+$CHCl_2$)$^+$ 54.02

Figure S11. Comparison of relative stabilities of [M+$CHCl_2$]$^+$ of butyrophenone (BuPh), 1-phenyl-2-butanone (1Ph-2Bu), and 4-phenyl-2-butanone (4Ph-2Bu). The relative energies are in kJ mol^{-1}. The higher stability of [M+$CHCl_2$]$^+$ ion of butyrophenone is in agreement with the MS spectra in which a sharp peak was observed for butyrophenone while no [M+$CHCl_2$]$^+$ peak was seen for other two isomers.

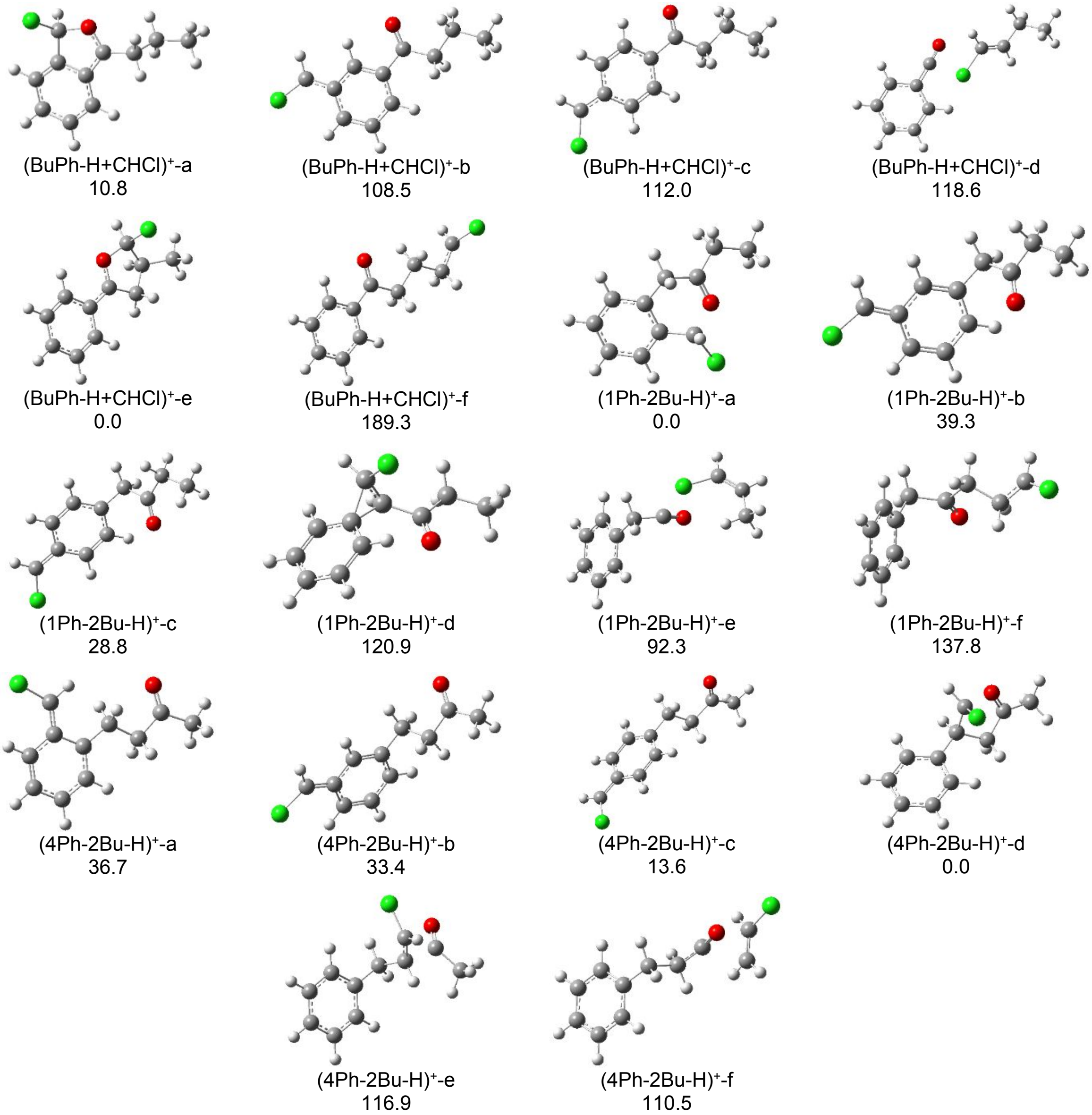

Figure S12. The optimized structures of different isomers of $[M-H+CHCl]^+$ for butyrophenone (BuPh), 1-phenyl-2-butanone (1Ph-2Bu), and 4-phenyl-2-butanone (4Ph-2Bu). The relative energies are in kJ mol^{-1}.

Table S2. Calculated ΔH and ΔG values for electrophilic substitution of $CHCl_2^+$ into butyrophenone (BuPh), 1-phenyl-2-butanone (1Ph-2Bu), and 4-phenyl-2-butanone (4Ph-2Bu). Only the most stable isomers have been considered.

**Reaction**	**ΔH (kJ mol^{-1})**	**ΔH (kJ mol^{-1})**
BuPh + $CHCl_2^+$ → $[BuPh-H+CHCl]^+$ + HCl	-303.2	-282.7
1Ph-2Bu + $CHCl_2^+$ → $[1Ph-2Bu-H+CHCl]^+$ + HCl	-268.0	-246.3
4Ph-2Bu + $CHCl_2^+$ → $[4Ph-2Bu-H+CHCl]^+$ + HCl	-249.8	-226.8

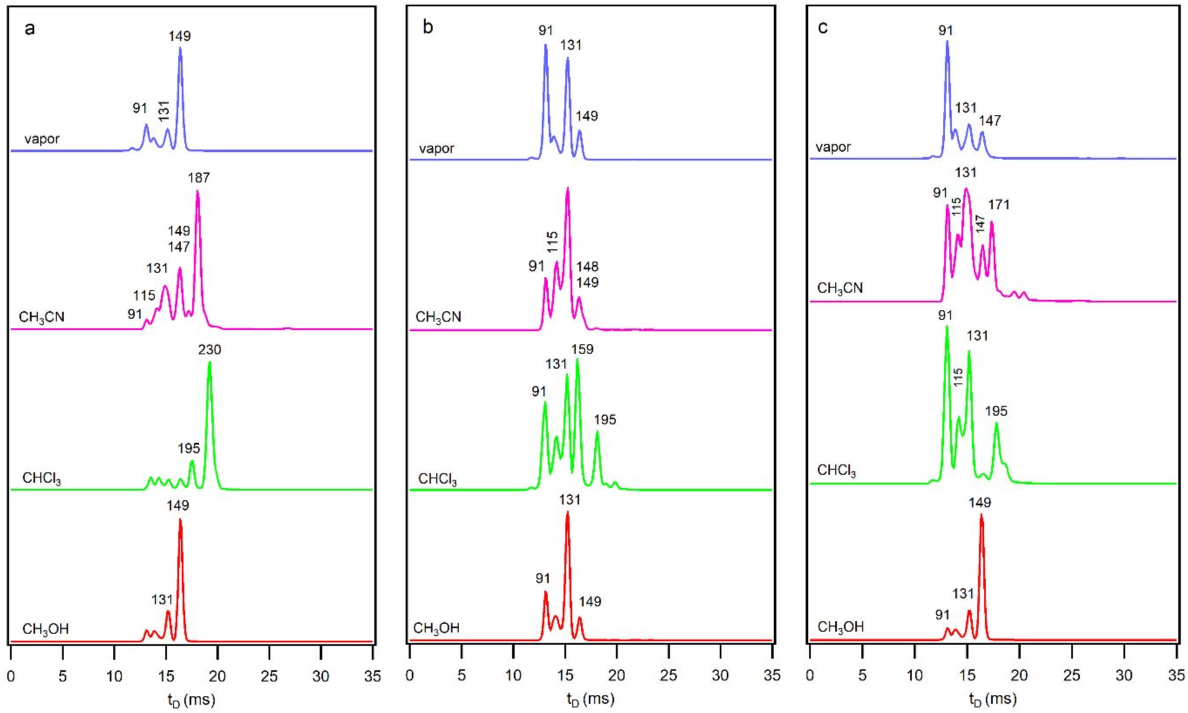

Figure S13. Total ion mobility spectra for the full mass range (*m/z*=50-240) of (a) butyrophenone, (b) 1-phenyl-2-buthanone, and (c) 4-phenyl-2-buthanone in in vapor and in solvents acetonitrile, chloroform and methanol with concentrations of 0.01%. The numbers above the peaks indicate their corresponding *m/z*.

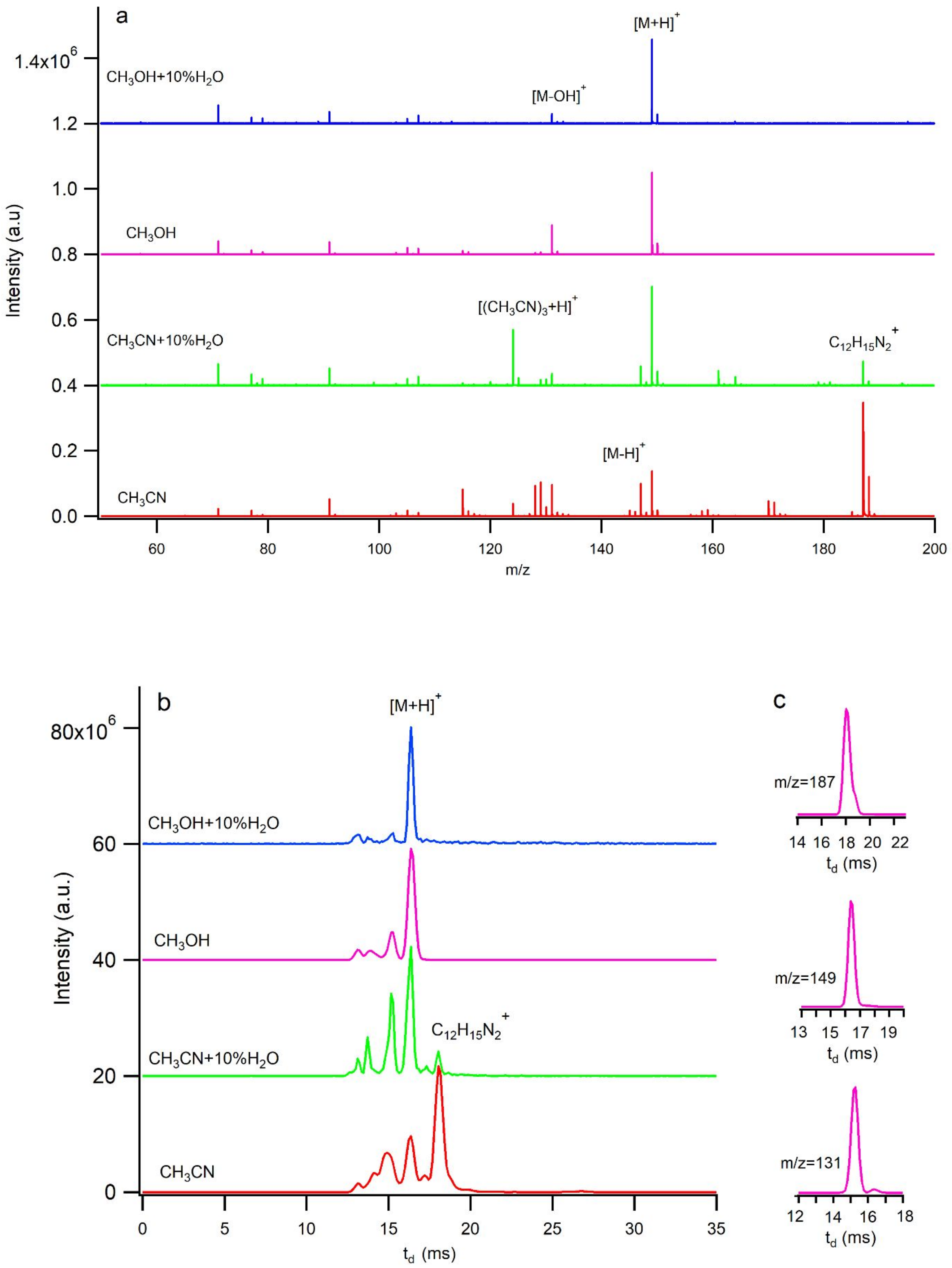

Figure S14. Comparison of (a) mass spectra and (b) total ion mobility spectra for the full mass range (*m/z*=50-240) of butyrophenone in acetonitrile, acetonitrile+10% water, methanol, and methanol+10% water. (c) The mass-selected ion mobility peaks for *m/z* of 187.1232, 149.0957, and 131.0853.

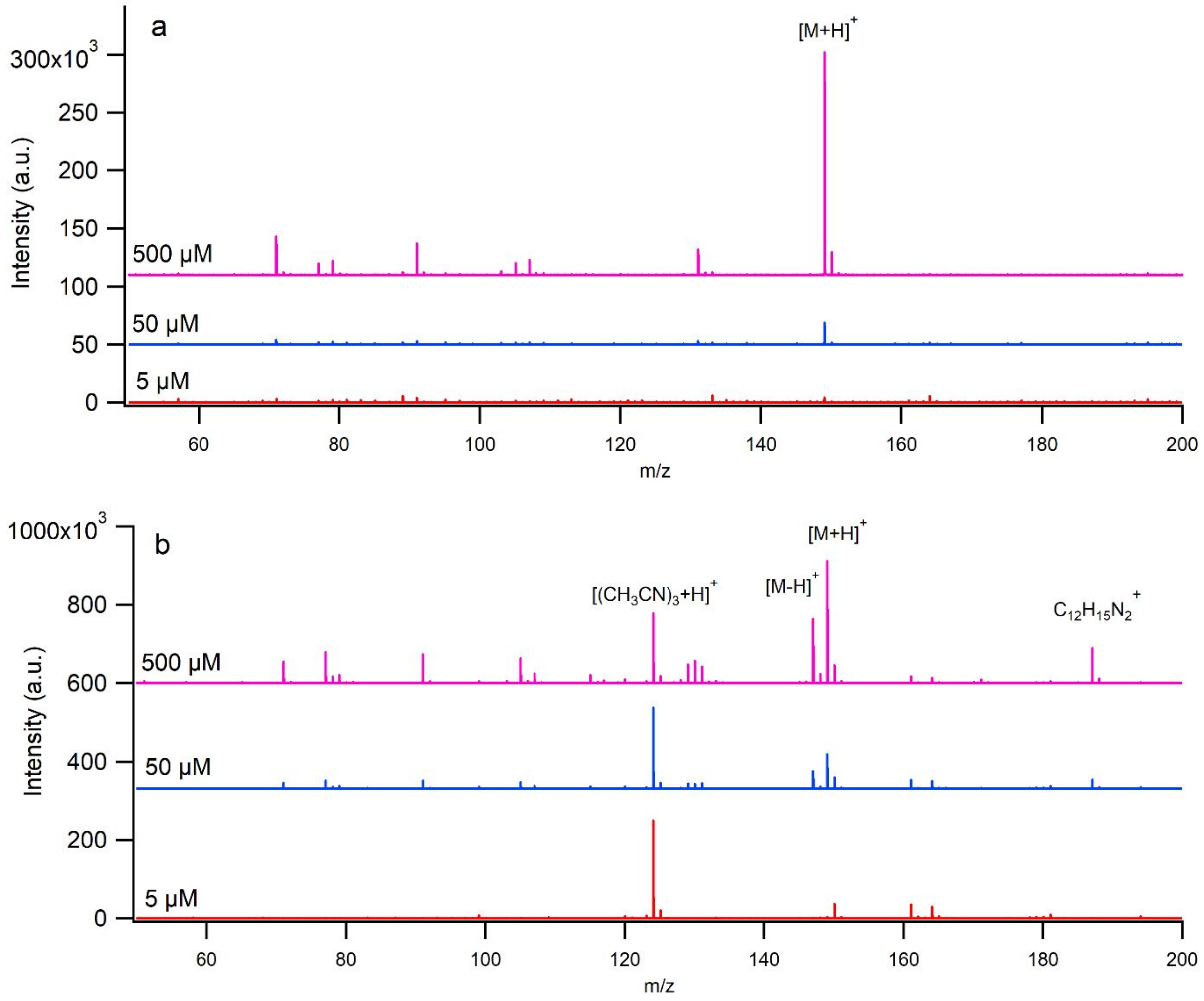

Figure S15. Comparison of APCI mass spectra of butyrophenone with different concentrations prepared in (a) methanol and (b) acetonitrile.

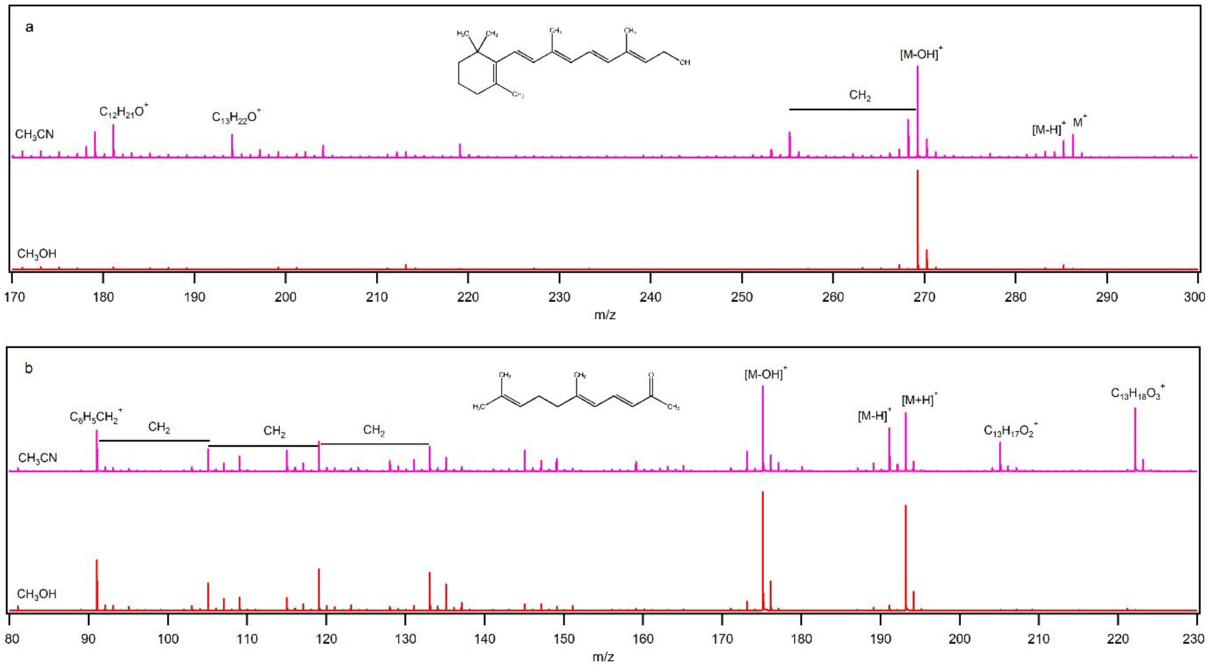

Figure S16. Comparison of mass spectra of (a) retinol and (b) pseudoionone prepared in acetonitrile and methanol solvents.

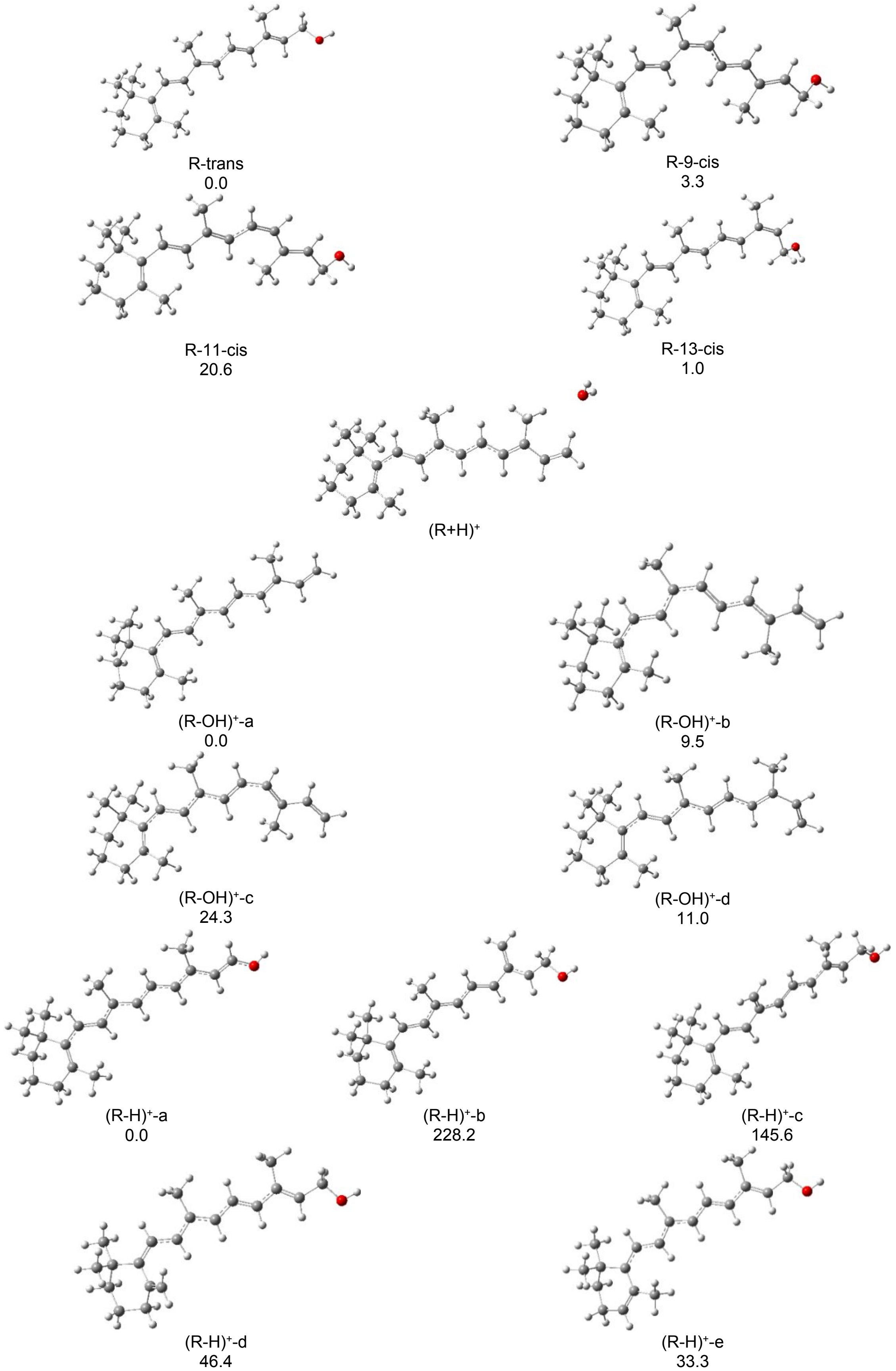

Figure S17. The optimized structures of different isomers of retinol (R), $[R+H]^+$, $(R\text{-}OH)^+$, and $[R\text{-}H]^+$. The relative energies are in kJ mol^{-1}.

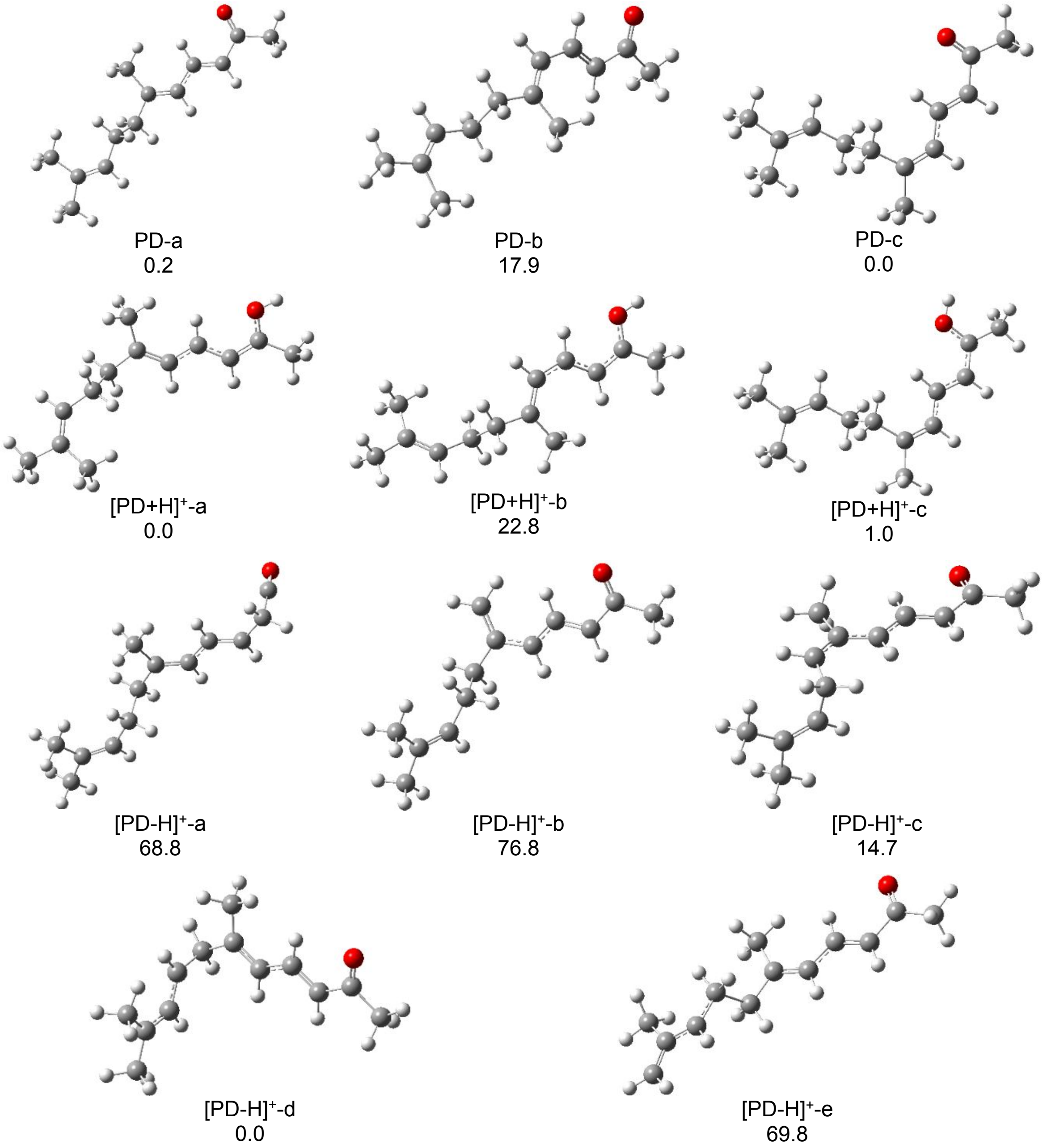

Figure S18. The optimized structures of different isomers of pseudoionone (PD), $[PD+H]^+$, and $[PD-H]^+$. The relative energies are in kJ mol^{-1}.

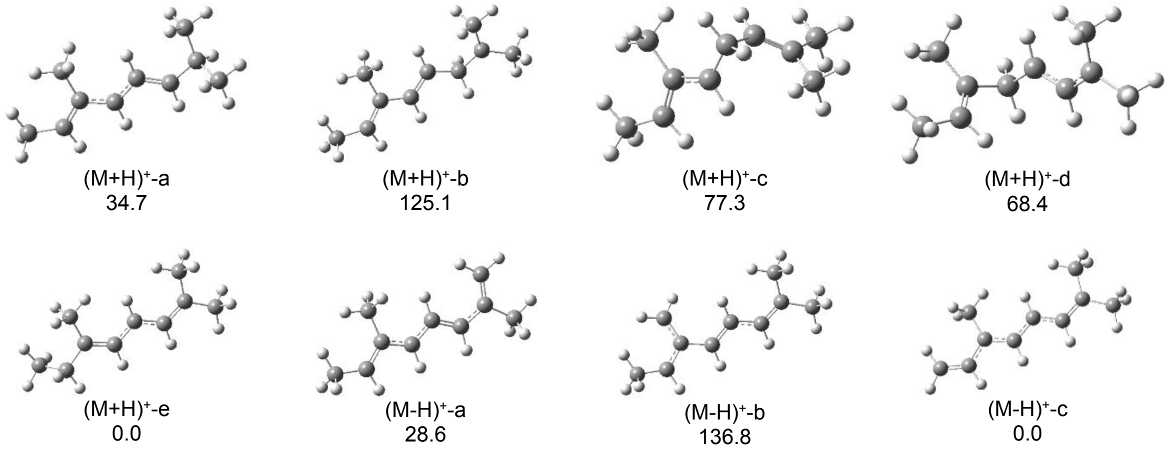

Figure S19. Optimized structures of different isomers for $[M+H]^+$ and $[M-H]^+$ ions of 2,6-dimethyl-2,4,6-octatriene. The relative energies are in kJ mol^{-1}.

Table S3. The measured *m/z* for the studied benzene derivatives and polycyclic aromatic hydrocarbons and their difference to theoretical masses (in ppm).

Compounds	Measured *m/z*	formula	Difference (ppm)
Benzene vapor, in Acetonitrile, in Methanol	78.0461	$C_6H_6^+$	-4.52
Benzene in Chloroform	125.0148	$C_7H_6Cl^+$	-4.46
Toluene vapor	91.0548	$C_7H_7^+$	6.84
Toluene Acetonitrile	91.0541	$C_7H_7^+$	-1.57
Toluene Chloroform	139.0321	$C_8H_8Cl^+$	4.85
	172.9915	$C_8H_7Cl_2^+$	-2.64
Toluene Methanol	91.0536	$C_7H_7^+$	-5.98
	97.0278	$C_5H_5O_2^+$	-6.23
	127.0381	$C_6H_7O_3^+$	-5.93
	143.0330	$C_6H_7O_4^+$	-5.95
Chlorobenzene vapor	112.0078	$C_6H_5Cl^+$	3.64
Chlorobenzene in Acetonitrile	112.0079	$C_6H_5Cl^+$	4.2
Chlorobenzene in Chloroform	112.0078	$C_6H_5Cl^+$	3.79
	158.9768	$C_7H_5Cl_2^+$	3.29
Chlorobenzene in Methanol	112.0079	$C_6H_5Cl^+$	3.61
Benzonitrile vapor	104.0489	$C_7H_6N^+$	-5.31
Benzonitrile in Acetonitrile	104.0496	$C_7H_6N^+$	1.33
	186.1030	$C_{11}H_{12}N_3^+$	2.41
Benzonitrile in Chloroform	104.0500	$C_7H_6N^+$	5.37
Benzonitrile in Methanol	104.0501	$C_7H_6N^+$	5.84
Tetracene in Acetonitrile	228.0944	$C_{18}H_{12}^+$	-1.43
	229.1006	$C_{18}H_{13}^+$	-1.43
Tetracene in Chloroform	228.0927	$C_{18}H_{12}^+$	-3.92
	229.1002	$C_{18}H_{13}^+$	-3.92
Tetracene in Methanol	229.1005	$C_{18}H_{13}^+$	-2.85
Pentacene in Acetonitrile	278.1094	$C_{22}H_{14}^+$	1.53
	279.1157	$C_{22}H_{15}^+$	-3.66
	309.0911	$C_{22}H_{13}O_2^+$	1.22
Pentacene in Chloroform	278.1081	$C_{22}H_{14}^+$	-3.99
	279.1156	$C_{22}H_{15}^+$	-3.99
Pentacene in Methanol	279.1157	$C_{22}H_{15}^+$	-3.17
Benzo[a]pyrene in Acetonitrile	252.0957	$C_{20}H_{12}^+$	4.03
	253.1021	$C_{20}H_{13}^+$	3.97
Benzo[a]pyrene in Chloroform	252.0956	$C_{20}H_{12}^+$	5.20
	253.1025	$C_{20}H_{13}^+$	5.18
Benzo[a]pyrene in Methanol	253.1021	$C_{20}H_{13}^+$	3.47

Table S4. The measured m/z for the studied phenyl butanone isomers and the polyenes and their difference with theoretical masses (in ppm).

**Compounds**	**Measured *m/z***	**formula**	**Difference (ppm)**
Common fragments of phenyl butanones	91.0545	$C_7H_7^+$	2.59
	105.0697	$C_8H_9^+$	-1.8
	115.0541	$C_9H_7^+$	-1.0
	129.0699	$C_{10}H_9^+$	0.19
	131.0853	$C_{10}H_{11}^+$	-1.84
Butyrophenone in Acetonitrile	149.0957	$C_{10}H_{13}O^+$	-2.47
	147.0805	$C_{10}H_{11}O^+$	1.37
	187.1232	$C_{12}H_{15}N_2^+$	1.24
Butyrophenone in Chloroform	195.0570	$C_{11}H_{12}ClO^+$	-0.8
	230.0496	$C_{11}H_{14}Cl_2N^+$	-0.59
	231.0339	$C_{11}H_{13}Cl_2O^+$	0.48
Butyrophenone in Methanol	149.0957	$C_{10}H_{13}O^+$	-2.77
1-Phenyl-2-butanone in Acetonitrile	149.0956	$C_{10}H_{13}O^+$	-3.24
	148.0881	$C_{10}H_{12}O^+$	-3.26
1-Phenyl-2-butanone in Chloroform	159.0810	$C_{11}H_{11}O^+$	3.17
	195.0571	$C_{11}H_{12}ClO^+$	0.22
1-Phenyl-2-butanone in Methanol	149.0957	$C_{10}H_{13}O^+$	-1.18
4-Phenyl-2-butanone in Acetonitrile	147.0804	$C_{10}H_{11}O^+$	1.83
	170.0959	$C_{12}H_{12}N^+$	-2.93
	171.1040	$C_{12}H_{13}N^+$	-1.62
4-Phenyl-2-butanone in Chloroform	195.0573	$C_{11}H_{12}ClO^+$	-0.17
4-Phenyl-2-butanone in Methanol	149.0969	$C_{10}H_{13}O^+$	4.91
Retinol in Acetonitrile	286.2282	$C_{20}H_{30}O^+$	-4.11
	285.2220	$C_{20}H_{29}O^+$	2.35
	269.2271	$C_{20}H_{29}^+$	2.25
Retinol in Methanol	269.2271	$C_{20}H_{29}^+$	3.35
	181.1229	$C_{11}H_{17}O^+$	3.24
Pseudoionone in Acetonitrile	193.1589	$C_{13}H_{21}O^+$	0.77
	191.1429	$C_{13}H_{19}O^+$	-0.18
	175.1476	$C_{13}H_{19}^+$	-4.46
Pseudoionone in Methanol	193.1594	$C_{13}H_{21}O^+$	4.12
	175.1488	$C_{13}H_{19}^+$	3.20
2,6-dimethyl-2,4,6-octatriene in Acetonitrile	121.1017	$C_9H_{13}^+$	5.05
	105.703	$C_8H_9^+$	4.6
2,6-dimethyl-2,4,6-octatriene in Methanol	137.1331	$C_{10}H_{17}^+$	3.72
